# Drug- and Vaccine-Induced Cutaneous T-Cell Lymphoma: A Systematic Review of the Literature

**DOI:** 10.1155/jskc/3103865

**Published:** 2025-02-27

**Authors:** Ifa Etesami, Mahshid Sadat Ansari, Elnaz Pourgholi, Sama Heidari, Arezou Rafati, Saeed Bahramian, Bardia Danaei, Sardar Demokri, Patrick Fazeli, Huria Memari, Hadis Mirzaee Godarzee, Bahar Sadeghi, Seyed Mohammad Vahabi

**Affiliations:** ^1^Department of Dermatology, Razi Hospital, Tehran University of Medical Sciences, Tehran, Iran; ^2^Department of Dermatology, Autoimmune Bullous Diseases Research Center, Tehran University of Medical Sciences, Tehran, Iran; ^3^Department of Dermatology, Skin Research Center, Shahid Beheshti University of Medical Sciences, Tehran, Iran; ^4^Department of Medicine, School of Medicine, Ilam University of Medical Sciences, Ilam, Iran; ^5^Department of Medicine, School of Medicine, Isfahan University of Medical Sciences, Isfahan, Iran; ^6^Department of Medicine, School of Medicine, Shahid Beheshti University of Medical Sciences, Tehran, Iran; ^7^Department of Medicine, School of Medicine, Urmia University of Medical Sciences, Urmia, Iran; ^8^Division of Biology & Medicine, Brown University, Providence, Rhode Island, USA; ^9^Department of Medicine, School of Medicine, Tehran University of Medical Sciences, Tehran, Iran

**Keywords:** cutaneous T-cell lymphomas, drug-induced, lymphomatoid papulosis, mycosis fungoides, subcutaneous panniculitis-like T-cell lymphoma, vaccine-induced

## Abstract

Cutaneous T-cell lymphomas (CTCLs) are a type of non-Hodgkin lymphoma that usually involves the skin. It has different subtypes including mycosis fungoides (MFs), Sézary syndrome (SS), primary cutaneous anaplastic large lymphoma (PC-ALCL), lymphomatoid papulosis (LyP), and subcutaneous panniculitis–like T-cell lymphoma (SPTCL). There are several reports of incidence, relapse, or progression of CTCLs by using specific drugs. We aim to identify drug- and vaccine-induced CTCL characteristics. A systematic search was conducted using MeSH terms/keywords: CTCL and drug-induced or drug-associated or vaccine-associated or vaccine induced through PubMed/Medline, Scopus, Web of Science, and Embase until May 10, 2024. Out of 14,031 papers, 60 articles were included, involving 71 patients with a mean age of 53.5 ± 17 years. Among them, 52.1% were male. Medications were categorized into four groups: conventional, biologics, small molecules, and vaccines. The most frequently reported medications in the first group were fingolimod (*n* = 8) and methotrexate (*n* = 7). Infliximab (*n* = 6) and etanercept (*n* = 5) were the most commonly reported biologics. Pfizer–BioNTech (*n* = 11) vaccine and JAK inhibitors (*n* = 3) were the most reported vaccine and small molecules. LyP (*n* = 17) was the most frequently reported type of CTCL, followed by PC-ALCL (*n* = 13), MF (*n* = 11), SS (*n* = 8), and SPTCL (*n* = 8). The most common underlying conditions were rheumatoid arthritis (*n* = 15) and multiple sclerosis (*n* = 10). Twenty patients (28%) experienced disease regression after discontinuing the drug, with a mean ± SD of 8.6 ± 8.8 weeks. In 14 patients (20%), chemotherapy and/or radiotherapy were initiated. Six patients passed away after being diagnosed with CTCL: two because of CTCL recurrence and four because of other complications. It is important recognizing CTCL as a possible, although rare, adverse effect of certain drugs and vaccines, and taking a history of vaccinations, especially COVID-19 vaccines, and immunosuppressive drugs such as fingolimod, TNF-a inhibitors, and methotrexate.

## 1. Introduction

Cutaneous T-cell lymphomas (CTCLs) are a type of non-Hodgkin lymphoma that usually involves the skin and rarely has extracutaneous invasion [1]. CTCL incidence increases by age, with a mean age of around 54 years, and it is not common in children [2, 3]. CTCL has different subtypes including mycosis fungoides (MFs), Sézary syndrome (SS), primary cutaneous anaplastic large lymphoma (PC-ALCL), lymphomatoid papulosis (LyP), subcutaneous panniculitis–like T-cell lymphoma (SPTCL), and extranodal NK/T-cell lymphoma. The most common subtypes are MF, PC-ALCL, and LyP with a portion about 80% of disease frequency [4].

Clinical presentation varies from single patch or plaques to generalized lesions or blood involvement. Because of the wide spectrum of manifestations, the diagnosis is based on clinical and histopathological features [1, 4]. Disease-specific survival depends on the subtype and varies from around 10%–100% [4]. However, it prominently decreases patients' quality of life [5]. Although there is not any curative treatment, there are several treatment options such as phototherapy, chemotherapy, systemic drugs, and stem cell transplantation [1].

CTCL pathogenesis is not completely discovered yet. Studies show that several factors including various mutations, different subsets of T-cells, chemokine receptors, and microRNA dysregulation and several molecular mechanisms such as JAK-STAT pathways are involved in disease incidence and progression [6, 7].

There are several reports of incidence, relapse, or progression of CTCLs by using specific drugs [8–11]. Although the exact mechanism is not clear yet, it seems this happens due to the dysregulation of specific pathways such as JAK-STAT, which have a role in disease pathogenesis. Other probable hypotheses are as follows: PD-1 inhibitors lead T-cell proliferation [12], roles of monoclonal antibodies by affecting interleukins [12], immunosuppression [13], and some other unclear immunological mechanisms [14].

The aim of this systematic review is to identify drugs and vaccines that were associated with the incidence of CTCLs, their course of disease, and outcomes through the literature.

## 2. Materials and Methods

This systematic review aims to clarify the drugs and vaccines that were reported to be associated with the incidence of CTCL, associated clinical characteristics, and course of disease. It follows the 2020 guidelines of the Preferred Reporting Items for Systematic Reviews and Meta-Analyses (PRISMA) [15].

### 2.1. Search Strategy

A systematic search was conducted using MeSH terms/keywords CTCL and, drug-induced or drug-associated or vaccine-associated or vaccine induced through PubMed/Medline, Scopus, Web of Science, and Embase until May 10, 2024 (Supporting file 1).

### 2.2. Eligibility Criteria and Study Selection

**Figure 1:**
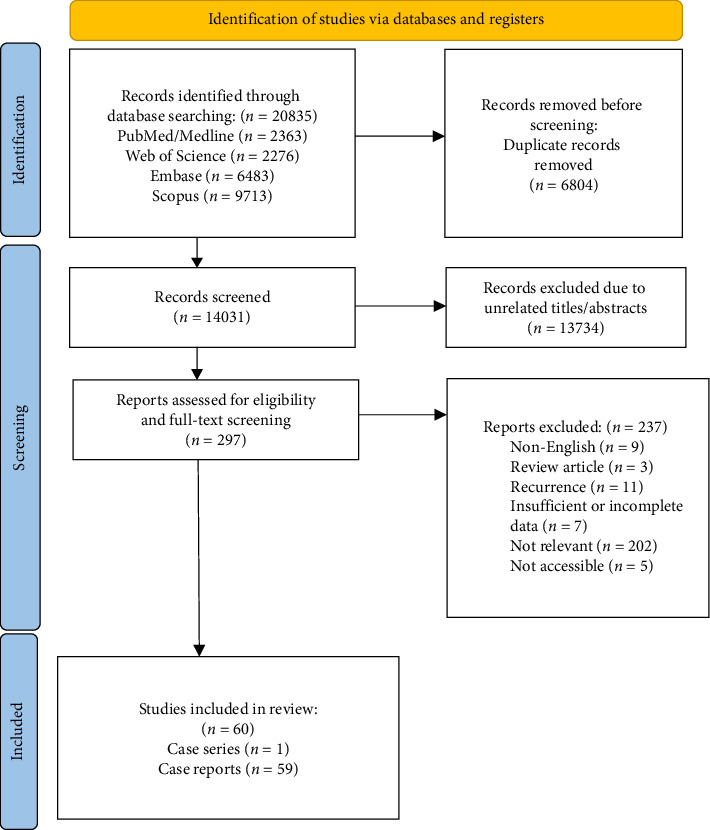
Preferred Reporting Items for Systematic Reviews and Meta-Analyses (PRISMA) flowchart of the number of studies identified and selected into the systematic review and meta-analysis.

This systematic review includes cross-sectional studies, case series, and case reports that reported new-onset CTCL disease after using a specific drug or vaccine. The were as follows: A diagnosed CTCL after receiving a specific drug or vaccine with enough evidence of that drug or vaccine triggered the CTCL. We excluded reviews, animal studies, cases that had a history of CTCLs or Hodgkin lymphoma, post-transplant cases, and cases with a phenotypic shift of disease. We excluded the cases of atopic dermatitis that showed CTCLs after using Dupilumab because of the possibility of CTCL misdiagnosed as atopic dermatitis and previous reviews in the literature on Dupilumab association with CTCLs [16] (Figure 1).

### 2.3. Data Extraction

Initially, eight reviewers independently screened the articles and excluded unrelated ones. In the case of disagreement, the corresponding author made the final decision. Data extracted included study characteristics, patient age, sex, underlying disease, main drug, onset of CTCL after using the drug, clinical features, histopathology, CTCL subtype, stage, management, and outcomes.

### 2.4. Naranjo Score

For the possibility of adverse drug reaction (ADR), we used the Naranjo score [17]. It consists of 10 questions scoring from −1 to +2, and the sum of scores (range −4 to +13) interprets as follows: Definite ≥ 9, Probable if 5–8, Possible if 1–4, and Doubtful ≤ 0.

## 3. Results

Out of initially 14,031 screened articles, 60 were included, comprising 59 case reports and one case series, involving 71 patients. The median (IQR) age of participants was 56 (42–69) and the mean (±SE) was 53.5 ± 2.05 years, ranging 13–84 years. Among the participants 52.1% were male. Medications were categorized into four groups: conventional treatments, biologics, small molecules, and vaccine-induced CTCLs.

### 3.1. Conventional Treatments

Twenty-eight articles and 30 patients were included [18–45]. The median (IQR) age of the patients was 56 (41–65), the mean (±SE) was 53.6 ± 2.9 years (ranging 13–84 years), and the median (IQR) time from drug intake to disease onset was 24 (7.25–108), with a mean (±SE) of 79.8 ± 26.9 months (approximately 5.8 years). The most common type of CTCL was PC-ALCL, with 11 cases (36.6%). Other frequently reported types included MF (*n* = 5, 16.6%), LyP (*n* = 5, 16.6%), SPTCL (*n* = 3, 10%), SS (*n* = 2, 6.6%), and primary cutaneous NK/T-cell lymphoma (*n* = 2, 6.6%). The most frequently reported medications were fingolimod (*n* = 8, 26.6%), methotrexate (MTX) (*n* = 7, 23%), and cyclosporine (*n* = 4, 13.3%) (Table 1).

### 3.2. Biologics

Twenty studies involving 22 individuals were analyzed [12, 46–64], with a median (IQR) age of 53 (41–69), mean (±SE) of 53.7 ± 3.6 years (21–81 years), median (IQR) onset of 18 (4.5–27.5), and an average (mean ± SE) of 23.6 ± 5.4 months (ranging from 2 weeks to 8 years). The most common type of CTCL identified was MF (*n* = 6, 27.3%), followed by SS (*n* = 4, 18.2%), SPTCL (*n* = 3, 13.6%), LyP (*n* = 2, 9%), and various other forms including nonspecified CTCL, primary aggressive cutaneous epidermotropic CD8 cytotoxic T-cell lymphoma, primary cutaneous gamma/delta T-cell lymphoma, primary cutaneous small/medium CD4+ T-cell lymphoma, cutaneous CD56+ T-cell lymphoma, cutaneous anaplastic large cell lymphoma, and cutaneous CD30+ T-cell lymphoma. Infliximab (*n* = 6, 27.3%), etanercept (*n* = 5, 22.7%), and adalimumab (*n* = 4, 18.2%) were the most commonly reported drugs in this classification (Table 2).

### 3.3. Vaccine

Eight studies involving 15 patients with new-onset CTCL were reported in this group [14, 65–71]. The median (IQR) age of the participants was 52 (30–66) and mean (±SE) was 50.6 ± 5.3 years (ranging from 18 to 80 years), and the median (IQR) and average (mean ± SE) onset of CTCL after drug initiation were 10 (4.75–22.5) and 14 ± 3.2 days (ranging from 3 to 42 days), respectively. The most common type of CTCL identified was LyP (*n* = 8, 53.3%), which included five cases of LyP Type A, one case of Type C, and one case of Type D. Other types included primary cutaneous small/medium CD4+ T-cell lymphoma (*n* = 3, 20%), SS (*n* = 1, 6.6%), primary cutaneous gamma/delta T-cell lymphoma (*n* = 1, 6.6%), SPTCL (*n* = 1, 6.6%), and one patient with PC-ALCL. Six out of the 15 patients (40%) presented with clinical manifestations following the first dose of COVID-19 vaccines. Five patients (33%) were referred after the second dose and four (27%) after the third dose. In total, 11 cases (73%) received the Pfizer–BioNTech vaccine, two received Moderna (13%), one received AstraZeneca (7%), and one patient (7%) received a Recombinant Adenovirus Type 26 vector-based vaccine (Table 3).

### 3.4. Small Molecule

Four studies included a total of four patients [72–75]. The median (IQR) age of the patients was 64 (45–77), with a mean (±SE) of 62 ± 8.4 years (ranging from 42 to 78 years), and the median (IQR) and average (mean ± SE) duration of disease onset were 3 (0.87–6.25) and 3.3 ± 1.4 months (ranging from 2 weeks to 7 months), respectively. Two cases (50%) were reported with LyP (one of which was Type A), while the remaining cases were diagnosed with primary cutaneous small/medium CD4+ T-cell lymphoma (25%) and SS (25%) (Table 4).

### 3.5. Drugs and CTCL

The most commonly associated drugs were TNFα-blockers (15/71, 21.1%), which included etanercept (*n* = 5), infliximab (*n* = 6), and adalimumab (*n* = 4). Other frequently reported medications included fingolimod (8/71, 11.2%), MTX (7/71, 9.8%), and cyclosporine (4/71, 5.6%). There were two cases each of melphalan, peginterferon, and pembrolizumab. Additionally, estradiol patch, goserelin, carbamazepine, phenytoin, glatiramer, hydrochlorothiazide, atenolol, benralizumab, nivolumab, tocilizumab, rituximab, and secukinumab were also reported. LyP was the most frequently reported type of CTCL (17/71, 23.9%) following drug initiation, comprising seven cases of LyP Type A, one case of Type B, one case of Type C, and two cases of Type D. The second and third most common types were PC-ALCL (13/71, 18.3%) and MF (11/71, 15.5%). Other common types of CTCLs included SS (8/71, 11.2%), SPTCL (8/71, 11.2%), and primary cutaneous small/medium CD4+ T-cell lymphoma (5/71, 7%).

The most common underlying conditions was rheumatoid arthritis, which accounted for 15 cases (21%) and was primarily treated with MTX in seven patients, biologics in six, and small molecules in two. Multiple sclerosis was present in 10 cases (14%), with fingolimod being the predominant treatment in eight cases. Melanoma was reported in six cases (8.5%), with five patients receiving biologic treatments, while psoriasis was noted in five cases (7%), with four individuals treated with biologics. Additionally, 14 cases (20%) did not have a documented past medical history, and nearly all of these patients, except for one, received COVID-19 vaccinations. Further details on the common underlying conditions associated with the most reported cases of CTCL are provided in Table 5.

Naranjo ADR Probability Scale is a questionnaire comprising 10 questions that assess the causality of adverse reactions associated with the drugs used in studies. Each question is assigned a score, and the total score is calculated by summing all individual scores. The final score indicates the probability of the adverse reaction as follows: ADR < 2 Doubtful, ADR 2–4 Possible, ADR 5–8 Probable, and ADR ≥ 9 Definite. The median (IQR) and average (mean ± SE) ADR were 4 (3.5–5) and 4.4 ± 0.2 (with a range of 2–8), respectively. Among the assessed drugs and vaccines, Pfizer–BioNTech COVID-19 vaccine exhibited the highest probability of adverse reactions, with a score of (mean ± SE) 5.5 ± 0.5, while adalimumab had the lowest probability, with a score of 3.5 ± 0.2 (Table 6).

Regarding the outcomes, a total of 20 patients (28%) experienced disease regression after discontinuing the drug, with a median (IQR) of 6 (1.75–13.25) and mean ± SE of 8.6 ± 2.5 weeks (ranging from 4 days to 32 weeks; six cases were not reported). However, this was associated with recurrence in one case. For 14 patients (20%), chemotherapy and/or radiotherapy were initiated. Most cases diagnosed with LyP did not receive any treatment (7/17, 41%). However, three cases (17%) were treated with MTX, and three other cases (17%) received phototherapy (two of which were treated with steroids and MTX). Additionally, two cases (12%) received topical steroids and intravenous steroids combined with antihistamines, while two cases (12%) did not have any treatments mentioned. Similarly, the majority of patients with ALCL also did not receive treatment after diagnosis (7/13, 54%), although three cases (23%) received radiotherapy. Other patients were treated with excision, topical steroids, and MTX. In the MF group, five individuals (45%) were treated with phototherapy, primarily in combination with other medications. Two cases (18%) received topical corticosteroids, and one case (9%) was treated with both radiotherapy and surgery. Two patients (18%) did not receive any treatment, and one case (9%) was not reported to have received any medications.

Six patients passed away after being diagnosed with CTCL. The cases owing to various causes of death varied and included lymphoma recurrence (in the cases of PC-ALCL and MTX-associated primary cutaneous extranodal NK/T-cell lymphoma), lung lymphoma (in the case of primary cutaneous CD8+ aggressive epidermotropic cytotoxic T-cell lymphoma), cardiovascular emboli (in case of SS), upper intestinal hemorrhage (in the cases of ALCL), and progression of underlying disease (in the case of primary cutaneous small/medium CD4+ T-cell lymphoma).

## 4. Discussion

This systematic review provided a comprehensive overview of the reported drug—and vaccine-associated CTCL cases. The findings contributed to the understanding of potential triggers and the clinical course of this rare but challenging disease. A total of 71 patients were reviewed with a mean age of 53.5 years, and a male predominance aligned with the known epidemiology of CTCL [2]. The most common associated drug was TNF inhibitors, accounting for 21% of cases, followed by fingolimod (11.2%). The most frequently reported subtype of CTCL was LyP, followed by PC-ALCL, MF, and SS. The shortest and longest time interval between receiving drugs/vaccine and onset of the CTCL was observed in the vaccines (mean 14 days) and conventional treatments (mean 79.8 months), respectively.

Several theories have been proposed to explain the pathophysiology of drug- and vaccine-associated CTCL. Immune system dysregulation is among the most widely recognized. The dysregulation of the JAK-STAT pathway, a key signaling mechanism in CTCL progression, is particularly evident in patients treated with immunomodulatory agents or biologics [6, 7, 9]. Findings from previous studies on PD-1 inhibitors, monoclonal antibodies, and other immune checkpoint modulators further support the idea that immune dysregulation underlies many cases [12, 13]. Notably, vaccine-associated cases predominantly occurred after COVID-19 vaccination, suggesting that immunological activation and/or T-cell–mediated responses to vaccine components, including mRNA might precipitate CTCL [14, 66, 70]. This highlights the complex interaction between therapeutic or prophylactic interventions and immune pathways in triggering CTCL.

Among the patients with underlying diseases, autoimmune conditions accounted for nearly two-thirds (37 out of 56 patients) when the vaccine group was excluded. Notably, all patients with autoimmune diseases had been treated with immunosuppressive or immunomodulatory drugs, except for one MS patient who developed CTCL after receiving a COVID-19 vaccine. This finding further supports the hypothesis that drug-associated CTCL may result from the immunosuppressive effects of treatment, the underlying immune dysfunction, or a combination of both factors.

Among the subtypes of drug-associated CTCLs, LyP was the most frequently reported in this review. Unlike its secondary rank in primary CTCL studies, this discrepancy may reflect the distinct immunological and molecular mechanisms underlying LyP [4, 76]. As CD30-positive lymphoproliferative disorder, LyP involves complex immunological and molecular mechanisms [77]. CD30, a tumor necrosis factor (TNF) receptor superfamily member, is expressed on activated T-cells [78]. When stimulated by antigens or mitogens, CD30 activation prevents apoptosis and promotes cell survival and proliferation, contributing to the pathogenesis of LyP [70, 79]. This review identified the COVID-19 vaccine as the most frequently associated trigger for LyP, followed by fingolimod, TNF inhibitors, and JAK inhibitors. These agents likely induce LyP through antigenic stimulation, consistent with its pathophysiology. Understanding this potential relationship emphasizes the need for clinical attention in patients developing LyP-like lesions after immunomodulatory therapies or vaccinations.

The second most commonly reported type of drug-associated CTCL in this review was PC-ALCL, another member of the CD30-positive lymphoproliferative disorder family [4, 78]. PC-ALCL and LyP represent the opposite ends of a spectrum of disorders with distinct prognoses, clinical presentations, and clinical courses in their classic forms. However, diagnosing borderline lesions can be challenging due to their overlapping histopathologic and immunophenotypic features [80, 81]. As previously discussed regarding the triggering role of medications in LyP pathophysiology, the shared mechanisms of PC-ALCL and LyP may clarify the frequent observation of PC-ALCL among drug-related CTCL subtypes. Notably, drugs such as cyclosporine, fingolimod, and methotrexate were commonly implicated, highlighting the need for vigilance in patients receiving these medications.

MF, the most common subtype of CTCL overall, ranked third in drug-associated cases [4, 76]. The pathogenesis and progression of MF are driven by a complex network of malignant mechanisms, primarily influenced by disrupted signaling pathways such as TCR/PLCγ1–NFAT, TNFR–NF-κB, and JAK–STAT [9, 82]. TNF inhibitors and fingolimod were the most frequently implicated agents. Similarly, in a previous study, the most commonly reported CTCL subtypes associated with biological agents were reported to be MF and SS [83]. The use of biologic agents such as TNF inhibitors and the resulting development of CTCL have been controversial in recent years [84, 85]. In our study of 71 drug-associated CTCL patients, 15 were treated with TNF-α inhibitors for a range of underlying autoimmune conditions, including psoriasis, psoriatic arthritis, Crohn's disease, ankylosing spondylitis, rheumatoid arthritis, and ulcerative colitis. Biologic therapy may be able to reveal an underlying lymphoma, particularly when taking into account the goal of biologic medicines to suppress both innate and acquired immunity, which regulate the growth of cancerous lymphocytes [83]. It remains unclear whether the development of CTCL is primarily driven by the immunosuppressive effects of the biologics or by the underlying immune disease itself; therefore, further studies are necessary to clarify this uncertainty.

Interestingly, fingolimod was the second most commonly associated drug in this study. Fingolimod, a sphingosine-1-phosphate receptor (S1PR) modulator, sequesters lymphocytes in lymph nodes and reduces their circulation in the bloodstream [86]. This altered trafficking can disrupt immune surveillance, potentially allowing malignant T-cell clones to evade detection and proliferate [87]. Furthermore, fingolimod's effects on S1PR signaling may facilitate the proliferation of malignant T-cell populations, which may also affect T-cell apoptosis and survival pathways [38]. These processes highlight a potential connection between the pathophysiology of CTCL and fingolimod.

The literature extensively documented the association between cyclosporine use and skin malignancies, particularly in organ transplant recipients [88, 89]. Additionally, cyclosporine achieved a high Naranjo ADR score due to the strong temporal relationship, exclusion of alternative causes, dose-dependent effects, and pre-existing evidence linking it to malignancies [17]. However, only four cases of cyclosporine-associated CTCLs were identified in this study. This lower-than-expected frequency could be explained by several factors. First, transplant patients were excluded from this review, which removed a substantial subset of individuals at heightened risk of cyclosporine-induced malignancies. Second, the most frequently reported malignancy associated with cyclosporine use is nonmelanoma skin cancer, whereas recent studies have not demonstrated an increased risk of lymphoma, including CTCL, among cyclosporine recipients. Furthermore, evidence from larger series indicates that cyclosporine treatment does not elevate lymphoma risk compared to azathioprine therapy [90]. These differences likely contribute to the lower observed incidence of CTCL in nontransplant patients.

The relatively rapid regression observed in six patients treated with fingolimod suggests that stopping fingolimod might play a significant role in disease resolution, highlighting its potential causal association with CTCL. In contrast, the data on MTX revealed considerable variability in regression intervals, potentially influenced by the CTCL subtype and other individual factors. Notably, disease regression following biologics tended to take longer, likely reflecting the prolonged immunomodulatory effects of these agents.

Persistent disease following drug cessation was noted in three patients who had received phenytoin, cyclosporine, and pembrolizumab. These patients developed SS, LyP Type A, and CD56+ T-cell lymphoma. The persistence of disease in these cases appears to be more closely related to the inherent nature of the CTCL subtype and the underlying disease rather than the specific drug.

The findings from the Naranjo ADR Probability Scale assessment offer important information about the possibility of drug-induced adverse events among the evaluated therapies [17]. An average score of 4.4 ± 0.2 in this study indicates that the assessed drugs fall into the “possible” category of causality. The Pfizer–BioNTech COVID-19 vaccine demonstrated the highest ADR probability (5.5 ± 0.5), which may be attributed to the unique immunologic mechanisms underlying vaccine-induced responses [70]. In contrast, adalimumab scored the lowest (3.5 ± 0.2), reflecting a relatively lower likelihood of adverse reactions. As a TNF inhibitor, its immunomodulatory effects are well-documented, and its safety profile has been extensively characterized, likely contributing to its lower ADR probability [85].

The clinical course and outcomes of drug- and vaccine-associated CTCL were notably heterogeneous across the reviewed studies. Approximately one-third of patients experienced significant improvement and disease regression upon cessation of the suspected triggering agent. This finding highlights the potential reversibility of CTCL in some cases when the associated drug or vaccine is withdrawn. However, one instance of recurrence after drug cessation suggests that the etiology of CTCL is likely multifactorial and influenced by underlying host factors [35]. Considering the final outcomes, the majority of patients in this review experienced disease regression, in line with findings reported in primary CTCL studies [1, 76, 91]. However, a subset showed poorer outcomes, with three patients (4.2%) experiencing recurrences and two (2.8%) exhibiting partial or no response to treatment. Among the six patients who passed away, three died from causes unrelated to CTCL, including metastatic melanoma, cardiovascular emboli, and upper gastrointestinal bleeding. However, the remaining three patients died due to lymphoma, highlighting the disease's severity, particularly in cases involving aggressive subtypes or advanced stages.

Among the patients with LyP, all achieved disease regression except for two cases of type D LyP, who experienced relapses. This finding aligns with primary CTCL studies demonstrating an almost 100% disease-specific survival rate over 10 years for LyP, reflecting its generally favorable prognosis [91]. Notably, Type D LyP, characterized by the pagetoid infiltration of small-to-medium atypical CD8+ epidermal cells, exhibits clinical behavior similar to other LyP subtypes [79]. Regarding PC-ALCL, all patients achieved disease regression without recurrence, except for one who passed away due to the extracutaneous involvement of the disease. This observation is consistent with the favorable prognosis reported in primary CTCL studies, where systemic dissemination occurs in approximately 10% of cases [91, 92]. Similarly, the outcomes for MF were comparable with those observed in primary cases [2, 76]. All patients experienced disease regression following drug discontinuation and received standard treatments according to their disease stage.

Treatment for drug- and vaccine-associated CTCL mirrored primary CTCL approaches [91, 93]. Chemoradiotherapy showed mixed outcomes [22, 47, 73], while phototherapy proved effective, particularly in the early stages [67, 69]. Other treatments, including corticosteroids, methotrexate, and surgical excision, had variable success, with some cases showing spontaneous regression [76, 79, 91, 93].

This review was limited by its reliance on case reports and small case series, which may introduce reporting bias and lack generalizability. However, ADRs usually are reported and even reviewing the reported cases is important to a better understanding of drug-induced CTCL. Additionally, one significant limitation of our study is the reliance on temporal associations to diagnose drug-induced CTCLs. While the onset of CTCL following drug initiation provides valuable diagnostic clues, it does not establish definitive causality, particularly in cases where the disease persists or recurs after drug cessation. This highlights the need for more robust diagnostic criteria and longitudinal studies to better understand the complex relationship between drug exposure and CTCL development. Future research should focus on larger cohort studies, mechanistic investigations, and prospective surveillance to elucidate the underlying immunological and molecular pathways. Genetic predisposition and environmental factors should also be considered potential contributors to susceptibility.

In conclusion, this systematic review emphasizes the importance of recognizing CTCL as a possible, although rare,

adverse effect of certain drugs and vaccines. Our review highlights the importance of taking a history of vaccinations, especially COVID-19 vaccines, and immunosuppressive drugs such as fingolimod, TNF-a inhibitors, and MTX in patients with a recent onset CTCL as a possible cause of disease.

## Figures and Tables

**Table 1:** Conventional-induced cutaneous T-cell lymphoma.

Study Year Gender/Age	Underlying diseases	Main drug	Onset of CTCL after medication CTCL subtype	Histopathology Immunophenotyping (IPT)	Management and outcome
Doyle et al. [18] 1994 M/73	Epilepsy, prostatic cancer	Phenytoin	60 years SS	Small lymphocytes with atypical mulberry nuclei, large lymphocytes with large hyperchromatic, convoluted nuclei, atypical blastic lymphocyte with cerebriform nuclei, Pautrier's microabscesses, Sézary cells IPT: CD2+, CD3+, CD4+, CD5+, CD8+, CD19−, CD20−, CD11c−, and TCRβ+	Drug cessation Disease persisted

Riyaz and Nair [19] 1999 F/74	HTN	Atenolol	2 years SS	Infiltration of atypical lymphocytes, histiocytes, and Sézary cells in the upper dermis IPT: −	Drug cessation

Di Lernia et al. [20] 2001 F/13	Lipothymic episodes	Carbamazepine	8 months PC-ALCL	Diffuse infiltration of large anaplastic lymphoid cells, moderate epidermotropism IPT: CD30+, ki-1 antigen, CD3+, CD43+, CD45RO+, and ALK-1−	Drug tapering and cessation, radiotherapy Disease regression after 4 months

Kirbya et al. [21] 2002 M/39	Eczema	Cyclosporine	2 years PC-ALCL	Large atypical cells with abundant cytoplasm and prominent nucleoli IPT: CD3+, CD30+, and EBER−	Drug cessation Disease regression within 6 months

Corazza et al. [22] 2003 F/61	Psoriasis	Cyclosporine	20 years PC-ALCL	Dense infiltration of large lymphocytes in the deep dermis IPT: CD8+, and CD30+	Drug cessation and radiotherapy Disease regressed four years later the lymphoma recurred and patient expired

Laube et al. [23] 2004 M/36	Atopic eczema, asthma, and allergic rhinitis	Cyclosporine	2 years LyP Type A	Polymorphic lymphocytes in the dermis with large cells, irregular nuclei and prominent nucleoli, mitoses, eosinophils, histiocytes, small lymphocytes IPT: LCA, CD3+, CD4+, CD43+, CD20+, CD79a+, CD30+, ALK-1−, EMA−, and EBER−	Dose reduction Disease persisted over years but regressed after MTX

Cox et al. [24] 2008 M/75	Prostatic cancer	Goserelin acetate injection	2 months CD30+ transformed MF stage: NA	Mild infiltration of atypical lymphocytes in the upper dermis, Pautrier microabscesses IPT: CD2+, CD3+, CD4+, CD30+, CD7−, CD8−, EMA-ALK-1−, and Ki67 > 70%	PUVA Lesion clearance without recurrence

Madray, Greene, and Butler [25] 2008 F/33	MS	Glatiramer acetate	4 months PC-ALCL	Diffuse infiltration of large atypical lymphocytes extending to subcutaneous tissue IPT: CD3+, CD30+, ALK−, EMA−, Ber-EP4−, and CD15−	Treatment was continued, and radiotherapy was added Disease regression

Parker, Solomon, and Lane [26] 2008 F/60	RA	MTX	12 years PC-ANKTCL	Minimal epidermotropism, infiltration of large atypical lymphocytes IPT: CD2+, CD3+, CD45+, CD4−, CD8−, CD5−, CD7-CD30−, CD20−, CD79a−, EBER+, TIA+, and TCR−	Drug cessation and radiotherapy Complete remission in 2 years

Nemoto et al. [27] 2010 M/60	RA	MTX	NA SPTCL	Diffuse infiltration of atypical lymphocytes in the subcutaneous adipose tissue, hyperchromatic nuclei IPT: CD3+, CD8+, EBER+, CD4−, CD20−, CD56−, and CD79a−	Drug cessation Disease regression within 15 months

Alloo, DeSimone, and Kupper [28] 2012 F/44	None	Estradiol patch	9 years MF, patch stage	Proliferation of CD4+ atypical lymphocytes, epidermotropism IPT: CD7−, and CD62L−	Potent topical corticosteroids Significant improvement

Peck, Frank, and Peck [29] 2013 F/56	Breast cancer, Hypercalciuria	Hydrochlorothiazide	7 months MF, Stage 1A	Infiltration of small to medium lymphocytes, epidermotropism IPT: TCRγ	Drug cessation, topical corticosteroids Disease regression

Gill et al. [30] 2014 M/50	Melanoma	Melphalan	18 years PC-ALCL	Atypical lymphocytes in the dermis IPT: CD30+, ALK−	Complete excision No recurrence within 4 years

F/56	Melanoma	Melphalan	4 years LyP	Perivascular infiltration of small and large lymphocytes in the superficial and mid-dermis IPT: CD3+ and CD30+	Follow-up No recurrence after 16 months

Samaraweera et al. [31] 2016 F/25	MS	Fingolimod	2 months LyP	NA IPT: CD30+	Drug cessation Disease regression after 6 weeks

Claudino et al. [32] 2016 F/66	RA, DM, HTN, P-HTN, and Kl-MCGP	MTX	NA PC-ALCL	Diffuse lymphocytic proliferation with focal necrosis, dermal edema, perivascular large atypical lymphoid cells IPT: CD3+, CD8+, CD30+, CD20−, CD56−, ALK−, EBER+, Granzyme B+, and TIA-1+	Drug cessation Disease regression

Cohen et al. [33] 2016 M/57	MS	Fingolimod	2 years LyP	NA IPT: NA	Drug cessation Disease regression after 6 weeks

Papathemeli et al. [34] 2016 F/53	MS, psoriasis	Fingolimod	3 months PC-ALCL	Infiltration of medium and large lymphocytes IPT: CD2+, CD3+, CD30+, CD56+, CD4−, CD7−, CD8−, CD15−, CD20−, Pax-5−, Granzyme B−, Ki67 65%, and EBER−	Drug cessation, topical corticosteroids Disease regression after 5 weeks

Matoula et al. [35] 2016 F/38	MS	Fingolimod	2 months LyP Type D	Diffuse, perivascular, dense infiltration of small, medium, and large lymphoid cells, epidermotropism, subepidermal edema IPT: CD3+, CD8+, and CD30+	Drug cessation Disease regression with recurrence

Nitsan et al. [36] 2018 F/53	MS	Fingolimod	3 years MF	Nodular lymphoid infiltrate with small irregular cells IPT: CD3+, CD20+, CD68+, and Ki67+	Drug cessation, radiotherapy, surgery Disease regression

Manouchehri et al. [37] 2018 F/42	MS	Fingolimod	2 years PC-ALCL	Diffuse infiltration of lymphoid cells in the dermis and subcutis, large and prominent nuclei IPT: CD3+, CD4+, CD30+, CD20−, CD10−, CD8−, CD7−, and Ki67 80%	Drug cessation Disease regression after 2 months

Froehlich et al. [38] 2018 F/44	MS	Fingolimod	4 months MF, Stage 2B (tumor stage)	Epidermotropic dermal infiltration of atypical CD4+T-cells IPT: NA	Drug cessation Disease regression after 4 weeks

Connolly et al. [39] 2018 F/51	MS	Fingolimod	10 months PC-ALCL	Dense dermal lymphoid infiltrate with large atypical CD30+ T-cells IPT: CD2+, CD3+, CD4+, ALK1−, CD8−, and CD30+	Drug cessation Disease regression within 2 months

Gambichler et al. [40] 2020 M/58	AD, AR, and Type I hypersensitivity	Cyclosporine	9 years PC-ALCL	Diffuse infiltration of large T-cells IPT: CD30+, EBER−, and TCR+	Drug tapering and cessation Disease regression after 4 days

Omori et al. [41] 2021 M/71	RA	MTX	6 years C-MTX-LPD	Infiltration of medium lymphocytes with atypical nuclei in the dermis IPT: CD3+, CD4+, CD30+, CD56+, Granzyme B+, EBER+, CD8−, CD20−, and ALK−	Drug cessation Disease regression after 4 months

Matsuoka et al. [42] 2022 F/65	RA	MTX	4 years SPTCL	Dense subcutaneous infiltration of small lymphocytes with rim formation around the adipocytes IPT: CD3+, CD4+, CD8+, TCR betaF1, TIA-1, Granzyme B, and perforin	Drug cessation Disease regression within a week

Sugimoto et al. [43] 2022 M/84	RA	MTX	15 years MTX-PCE-NKTCL	Perivascular infiltration of medium- to large-sized lymphocytes in the deep dermis with fat necrosis IPT: CD3+, CD30+, CD56+, EBER+, EBV LMP1, Granzyme B+, TCR-delta+, CD4−, CD8−, CD20−, EBNA2−, IMP3−, PAX5−, and TCR-beta F1-	Drug cessation and topical corticosteroids After 8 months patient expired due to recurrence

Masuda et al. [44] 2023 M/72	RA	MTX	10 years PC-ALCL	Diffuse infiltration of large, atypical lymphoid cells with irregular nuclei and prominent nucleoli, scattered Reed–Sternberg-like cells IPT: CD3+, CD4+, CD25+, CD30+, TIA-1+, CD8−, CD15−, CD20−, CD56−, ALK−, and EBER−	Drug cessation Disease regression within a week

Magro et al. [45] 2024 F/61 F/40	PV	Peginterferon Alfa-2a	13 months SPTCL	Infiltration of small- to medium-sized atypical lymphocytes into fat tissue, disruption of adipocyte cytoplasmic membrane, and internal rimming of the adipocyte membrane IPT: CD2+, CD3+, CD7+, CD8+, CD5−, CCR4, Ki67 30%, beta-f1+, and TCRγ-	Drug cessation and romidepsin administration Disease regression
	PV	Peginterferon alfa-2a	21 months SPTCL	Diffuse infiltration of small, medium, and large lymphocytes with nuclear irregularity into fat lobules, disruption of adipocyte cytoplasmic membrane IPT: CD2+, CD3+, CD7+, CD8+, CD5−, CCR4, Ki 67 15%, beta-f1+, and TCRγ-	Drug cessation Significant but not complete improvement

*Note:* PUVA: Psoralen plus ultraviolet-A, Kl-MCGP: kappa light-chain restricted monoclonal gammopathy, C-MTX-LPD: cutaneous methotrexate-related T-cell lymphoproliferative disorder, MTX-PCE-NKTCL: MTX-associated primary cutaneous extranodal NK/T-cell lymphoma.

Abbreviations: AD, atopic dermatitis; AR, actinic reticuloid; DM, diabetes mellitus; HTN, Hypertension; LyP, lymphomatoid papulosis; MF, mycosis fungoides; MS, multiple sclerosis; MTX, methotrexate; PC-ALCL, primary cutaneous anaplastic large-cell lymphoma; PC-ANKTCL, primary cutaneous atypical NK-/T-cell lymphoma; P-HTN, pulmonary hypertension; PV, polycythemia vera; RA, rheumatoid arthritis; SPTCL, subcutaneous panniculitis-like T-cell lymphoma; SS, Sézary syndrome.

**Table 2:** Biologics-induced cutaneous T-cell lymphoma.

Study Year Gender/age	Underlying diseases	Main drug	Onset of CTCL after medication CTCL subtype	Histopathology Immunophenotyping (IPT)	Management and outcome
Mahe et al. [46] 2003 M/47	Erythrodermic psoriasis	Infliximab	3 months Cutaneous CD30+ T-cell lymphoma	Large atypical nonepidermotropic lymphocytes, high mitotic index IPT: CD3+, CD4+, CD5+, CD30+, EBER+ CD8−, CD20−, and CD79a−	Drug cessation Disease regression

Adams et al. [47] 2004 M/69 F/81	Psoriatic arthritis	Etanercept	18 months SS	Epidermotropism, IPT: CD30+	Cyclophosphamide, vincristine, prednisone, chemotherapy initiated Patient died due to cardiovascular emboli within 3 months
	Crohn's disease	Infliximab	5 months PC-ALCL	Superficial and mid-dermal infiltrate of large anaplastic cells IPT: CD30+, CD20−, ALK−, deletion of CD3, 5, and 7, co-expression of CD71, and HLA-DR	MTX was initiated Patient died due to upper intestinal hemorrhage within 2 weeks

Dalle et al. [48] 2005 M/40	Ankylosing spondylitis	Adalimumab	7 months MF with follicular mucinosis	Perivascular infiltrate of follicular epithelium by medium-sized atypical T-cell, mucinous degeneration, no epidermotropism IPT: CD3+, and CD4+ TCR:*γ* chain	Treatment with retinoids and PUVA improvement

Dauendorffer et al. [49] 2006 M/75	Ankylosing spondylitis	Infliximab	16 months SS	Dermal infiltration of lymphocytes, mild lymphocyte exocytosis, atypical lymphocytes IPT: CD2+, CD3+, CD4+, CD5+, CD45RO+, and CD4/CD8 ratio: 51	Drug cessation + MTX Disease regression

Koens et al. [50] 2009 M/69	RA	Etanercept	3 years PCGD-TCL	Infiltration of medium-sized lymphocytes with pleomorphic nuclei in the subcutaneous fat and upper dermis, atypical lymphocytes around adipocytes, histiocytes IPT: CD2+, CD3+, CD5+, TIA-1 and Granzyme B, and EBER−	Drug cessation + increased dexamethasone dosage Disease regression

Lourari et al. [51] 2009 F/74 F/69	RA	Etanercept	2 months Pilotropic MF with follicular mucinosis Stage: NA	Lymphocyte infiltration with mucin degeneration around hair follicle IPT: EBER−	Drug cessation, phototherapy Resolution
	RA	Infliximab	29 months MF, stage: NA	Lymphocyte infiltration with band-like pattern and epidermotropism IPT: EBER−	Drug cessation Spontaneous regression

Michot et al. [52] 2009 F/50	RA	Etanercept	5 years SPTCL	Infiltration of small, atypical lymphoid cells around adipocytes occasional hemophagocytosis IPT: *α*/β+, CD3+, CD8+, CD56, TIA-1+cytotoxic T-cell, and EBER−	Drug cessation, chemotherapy Disease regression after 3 months

Outlaw, Fleischer, and Bloomfeld [53] 2009 M/21	Crohn's disease	Infliximab	2 years LyP	NA IPT: CD30+	Drug cessation Disease regression over a few months

Sluzevich, Hall, and Roy [54] 2012 M/76	CLL and hemolytic anemia	Rituximab	2 weeks SPTCL	Lobular panniculitis of atypical lymphocytes rimming adipocytes with fat necrosis IPT: CD3+, CD8+, TIA-1+, βF-1+, CD4−, CD30−, CD56−, and EBER−	Drug cessation Disease regression after 8 months

Bittencourt et al. [55] 2013 F/48	HTLV-1, SA, uveitis, bilateral episcleritis, neurogenic bladder, and severe eczema in childhood	Adalimumab	27 months MF; stage: NA	Pagetoid epidermotropism, infiltration of small- and medium-sized atypical lymphocytes IPT: CD3+, CD4+, CD8−, CD20−, CD25+, CD30− and CD68−, and Ki-67:8%	Phototherapy and topical corticosteroids No response Interferonα and zidovudine Complete response

Jacks et al. [56] 2014 F/35	Psoriasis	Adalimumab	2 years PCAECyTCL	Epidermotropic infiltrate of atypical CD8+ cells IPT: CD3+, CD8+, Granzyme B, and TIA-1, Ki-67, CD56+, and EBER−	The patients expired due to lung lymphoma

Ma et al. [57] 2014 M/62	RA	Etanercept	3 months PCSM-TCL	Diffuse infiltration of small- to medium-sized lymphocytes with mild atypia affecting eccrine glands IPT: CD2+, CD3+, CD4+, CD5+, CD7+ and CD45RO+, CD68+, CD20+, CD79a+, CD8+, CD30−, CD56−, TIA-1−, CD23− and Granzyme B−, EBER−, and TCR−	Drug cessation Spontaneous resolution of lesions

Nakamura et al. [58] 2017 F/21	Juvenile idiopathic arthritis	Tocilizumab	7 years SPTCL	Lobular panniculitis-like feature with necrotic debris and atypical lymphoid cells, rimming adipocytes IPT: CD3+, CD8+, Granzyme B+, and α/β T-cell receptor	Drug cessation Disease regression with no recurrence

Zheng et al. [12] 2018 M/61	Melanoma	Pembrolizumab	1 year C(CD56+)-TCL	Small lymphocytes, epidermotropism IPT: CD56+/TIA-1+/CD3+/bF1+/CD7+, CD4+, CD8+, EBER−, GM3−, CD123−, and PD-1-	Drug cessation, radiotherapy, pralatrexate No response

Szakonyi et al. [59] 2019 M/38	Crohn's disease	Adalimumab	18 months LyP Type B	Perivascular and periadnexal infiltration of small to medium lymphoid cells with epidermotropism IPT: CD3+, CD4+, CD30+, and TCRγ	Drug cessation, oral budesonide, topical corticosteroid, phototherapy Disease regression without recurrence

Cortonesi et al. [60] 2020 M/42	Psoriasis and psoriatic arthritis	Secukinumab	2 years SS	Parakeratosis, spongiosis, lymphohistiocytic infiltrate with eosinophil, perivascular, and subepidermal granulocytes, exocytosis IPT: CD4+, CD5+, CD30−, CD4+/CD8+, TCR-β, and TCR-γ	Extracorporeal photopheresis and bexarotene Disease regression within 4 months

Shin et al. [61] 2020 F/56	Melanoma	Pembrolizumab and ipilimumab	1 year MF	Infiltration of small- to medium-sized lymphocytes, epidermotropism with hyperchromatic nuclei IPT: CD2+, CD3+, CD5+, CD8+, TCR-betaF1, CD30+, PD-1, CD7−, CD4−, EBER−, Ki67 30%–40%, EBER−, TCR-β, and TCR-γ	NA

Yasuda et al. [62] 2020 M/46	Ulcerative colitis	Infliximab	8 years MF, stage: Infiltration stage	Infiltration of large atypical lymphocytes IPT: CD3+, CD5+, CD8+, and CD4−	Drug cessation, diflorasone, and phototherapy Disease regression during 1 year

Barre et al. [63] 2022 M/74	Asthma	Benralizumab	1 month SS	Perivascular infiltration of atypical lymphocytes, irregular nuclear contours, mild epidermotropism IPT: CD4+, CD7+, CD26−, and CD158k+	Extracorporeal photopheresis and MTX Complete response of skin and partial blood response

Hida et al. [64] 2022 M/56	Melanoma	Nivolumab	1.5 years Cutaneous T-cell lymphoma	Infiltration of atypical lymphocytes (panniculitis-like pattern) IPT: CD3+, CD8+, CD4−, CD56−, and EBER+	Chemotherapy Disease regression

*Note:* PUVA: psoralen plus ultraviolet-A, SPTCL: subcutaneous panniculitis-like T-cell lymphoma, PCAECyTCL: primary cutaneous CD8+ aggressive epidermotropic cytotoxic T-cell lymphoma, PCSM-TCL: primary cutaneous CD4+ small/medium-sized T-cell lymphoma.

Abbreviations: C(CD56+)-TCL, cutaneous CD56+ T-cell lymphoma; CLL, chronic lymphocytic leukemia; HTLV-1, human T-lymphotropic virus 1; LyP, lymphomatoid papulosis; MF, mycosis fungoides; MTX, methotrexate; PC-ALCL, primary cutaneous anaplastic large cell lymphoma; PCGD-TCL, primary cutaneous gamma-delta T-cell lymphoma; RA, rheumatoid arthritis; SA, spondyloarthritis; SS, Sézary syndrome.

**Table 3:** Vaccine-induced cutaneous T-cell lymphoma.

Study Year Gender/Age	Underlying diseases	Vaccine type	Onset of CTCL after vaccine Dose CTCL subtype	Histopathology Immunophenotyping (IPT)	Management and outcome
Avallone et al. [65] 2022 F/80 M/60 F/52 F/61 M/45	None	Pfizer–BioNTech COVID-19 vaccine SS	15 days 3rd dose	NA IPT: NA	Topical and oral corticosteroid Complete response
	None	Pfizer–BioNTech COVID-19 vaccine LyP Type A	30 days 2nd dose	Perivascular and interstitial infiltrate in the superficial dermis of small-sized and large, lymphocytes, numerous neutrophils, eosinophils, and histiocytes IPT: CD3+, CD4+, CD7+, CD30+, and GATA3+	IV corticosteroid and antihistamine Complete response
	NA	Pfizer–BioNTech COVID-19 vaccine PCSM-TCL	3 days First dose	Diffuse dense infiltrate in the dermis, small- to medium-sized hyperchromatic lymphocytes. Atypical lymphocytes IPT: CD4+, PD1+, and GATA3+	Radiotherapy Complete response
	NA	Pfizer–BioNTech COVID-19 vaccine LyP Type A	10 days First dose	Perivascular infiltrate in the superficial dermis of medium-/large-sized lymphocytes, eosinophils, and blast-cells IPT: CD3+, CD4+, CD7+, CD30+, and GATA+	Spontaneous resolution
	NA	Pfizer–BioNTech COVID-19 vaccine PCSM-TCL	20 days Third dose	NA IPT: NA	Surgical excision Complete resolution

Hooper et al. [66] 2022 M/50 F/20	MS	Pfizer–BioNTech COVID-19 vaccine LyP Type A	4 days First dose	Predominant infiltration of T lymphocytes and CD30+ atypical cells IPT: NA	NA
	NA	Pfizer–BioNTech COVID-19 vaccine LyP Type A	42 days Second dose	Predominant infiltration of T lymphocytes IPT: NA	NA

Koumaki et al. [67] 2022 M/60 F/66	Dyslipidemia, HTN	AstraZeneca (AZD1222) LyP	7 days First dose	Diffuse infiltration of polymorphic small and large lymphocytes and histiocytes IPT: CD4+, CD8+, and CD30+	Spontaneous regression within 3 months
	NA	Pfizer–BioNTech COVID-19 vaccine LyP Type D	10 days 2nd dose	Dense perivascular infiltration of small, medium, and large atypical lymphocytes, epidermotropism IPT: CD3+, CD8+, and CD30+	NB-UVB Disease recurrence after 3 months

Kreher et al. [68] 2022 F/28	NA	Adenovirus Type 26 viral vector-based COVID-19 vaccine SPTCL	Within few days First dos	Lobular panniculitis, infiltration of atypical lymphocytes around adipocytes with fibrinoid necrosis and large histiocytes with apoptotic debris IPT: CD31, CD71, CD81, Perforin 1, CD4−, CD56−, EBER−, ISH−, and CD30−	Cyclosporine and prednisolone Disease regression

Revenga-Porcel, Peñate, and Granados‐Pacheco [14] 2022 M/76	NA	Moderna PC-ALCL	10 days Third dose	Diffuse dermal infiltration of large lymphocytes, eosinophilic cytoplasm, large irregular nuclei, pleomorphism, and multiple nucleoli IPT: CD2+, CD4+, CD30+, CD5+, MUM1, Granzyme B+, CD3−, CD7−, and CD45−	Spontaneous remission

Ceravalls et al. [69] 2023 M/30	NA	Pfizer–BioNTech COVID-19 vaccine LyP Type A	5 days 2nd dose	Epidermotropism, large anaplastic cells, and small lymphocytes, neutrophils and eosinophils, atypical lymphocytes IPT: CD4+, and CD30+	NB-UVB: Partial response NB-UVB + MTX: Complete response

Gordon et al. [70] 2023 M/18 M/35	NA	Pfizer–BioNTech COVID-19 vaccine LyP Type C	1 month 2nd dose	Atypical, large lymphocyte, eosinophils, neutrophils, and histiocytes IPT: CD2+, CD3+, CD5+, CD7−, CD30+, CD4 dominance over CD8, CD21+, CD23+, and EBER−	MTX Complete remission
	NA	Pfizer–BioNTech COVID-19 vaccine PCSM-TCL	1 week 1st dose	Perivascular and adnexal infiltration of small- and medium-sized atypical noncerebriform lymphocyte without epidermotropism IPT: CD3+, CD4+, BCL6+, and PD1+	Follow-up Spontaneous resolution

Hobayan and Chung [71] 2023 M/79	NA	Moderna PCGD-TCL	3 days 3rd dose	Diffuse infiltration of atypical lymphocytes in the dermis and subcutis with numerous bean-bag cells, angiotropism, and angiodestruction IPT: CD3+, Granzyme B, CD4−, CD5−, CD7−, CD8−, ALK−, CD30−, and EBER−	Conservative and localized radiotherapy Disease regression

*Note:* PCSM-TCL: primary cutaneous CD4+ small/medium-sized T-cell lymphoma, SPTCL: subcutaneous panniculitis-like T-cell lymphoma.

Abbreviations: HTN, hypertension; LyP, lymphomatoid papulosis; MS, Mmultiple sclerosis; MTX, methotrexate; NB-UVB, narrowband ultraviolet B; PC-ALCL, primary cutaneous anaplastic large cell lymphoma; PCGD-TCL, primary cutaneous gamma-delta T-cell lymphoma; SS, Sézary syndrome.

**Table 4:** Small molecule-induced cutaneous T-cell lymphoma.

Study Year Gender/age	Underlying diseases	Main drug	Onset of CTCL after medication CTCL subtype	Histopathology Immunophenotyping (IPT)	Management and outcome
Garrido et al. [72] 2015 M/54	Melanoma	Vemurafenib	4 months PCSM-TCL	Lymphoid proliferation in the reticular dermis, small- to medium-sized and few large lymphocytes, atypic cells with hyperchromic nuclei IPT: CD3+, CD4+, CD8+, CD43+, PD-1, BCL-6, CD20 and CD79a	Surgery and radiotherapy, then drug cessation Patient expired due to underlying disease progression

Inuma et al. [73] 2022 F/74	RA	Upadacitinib	2 weeks LyP Type A	Atypical large lymphocytes in the superficial and mid-dermis, irregular nuclei, frequent mitoses, eosinophils, histiocytes, and small lymphocytes IPT: CD3+, CD4+, and CD30+	Drug cessation, topical corticosteroids Resolution of the lesions within 1 month

Knapp et al. [74] 2022 M/42	EED, arthritis	Tofacitinib	8 weeks LyP	Nodular infiltrate in the upper dermis, with increased numbers of large atypical CD30+ cells, small lymphocytes, neutrophils, eosinophils, histiocytes IPT: CD3+, CD4+, CD8+, CD20+, and EBER−	Drug cessation, MTX 80% clearance within 12 weeks

Saito et al. [75] 2023 M/78	RA	Baricitinib	7 months SS	Band-like infiltration of small- to medium-sized atypical lymphocytes, epidermotropism IPT: CD3+, CD4+, CCR4+, CCR7+, CD7−, and CD26−	Drug cessation, mogamulizumab, bexarotene, and phototherapy Partial response

*Note:* PCSM-TCL: primary cutaneous CD4+ small/medium-sized T-cell lymphoma.

Abbreviations: EED, erythema elevatum diutinum; LyP, lymphomatoid papulosis; MTX, methotrexate; RA, rheumatoid arthritis; SS, Sézary syndrome.

**Table 5:** Most frequent diseases associated with drugs and underlying diseases.

Total number: 62	LyP *N* = 17	PC-ALCL *N* = 13	MF *N* = 11	SS *N* = 8	SPTCL *N* = 8	PCSM-TCL *N* = 5
Gender (*n*, %)						
F	7 (41.2%)	8 (61.5%)	8 (72.7%)	2 (25%)	6 (75%)	1 (20%)
M	10 (58.8%)	5 (38.5%)	3 (27.3%)	6 (75%)	2 (25%)	4 (80%)

Age (mean ± SE) Median (IQR)	44.2 ± 4.2 42 (27.5–60)	53.4 ± 5.2 53 (40–69)	55 ± 3.7 53 (44–69)	70.6 ± 4.2 74 (70–77)	50.1 ± 6.7 55 (31–64)	49.6 ± 4.5 52 (40–58)

Medications	Pfizer–BioNTech COVID-19 vaccine (*n* = 7, 41%) Fingolimod (*n* = 3, 18%) TNFα-blockers (*n* = 2, 12%) JAK inhibitors (*n* = 2, 12%)	Cyclosporine (*n* = 3, 23%) Fingolimod (*n* = 3, 23%) MTX (*n* = 2, 15%)	TNFα-blockers (*n* = 5, 45%) Fingolimod (*n* = 2, 18%)	TNFα-blockers (*n* = 2, 25%) Other Biologics (*n* = 2, 25%)	Biologics (*n* = 3, 37.5%) MTX (*n* = 2, 25%) Peginterferon alpha-2a (*n* = 2, 25%)	Pfizer–BioNTech COVID-19 vaccine (*n* = 3, 60%)

Interval between drug initiation and CTCL onset (months, mean ± SE, median (IQR))	8.7 ± 3.3 1.4 (0.3–21)	63.5 ± 25.1 17 (4.25–117)	30 ± 11.3 12 (4–36)	101.3 ± 88.4 17 (2.5–24)	37.7 ± 12.9 34 (9.85–66)	1.5 ± 0.8 0.6 (0.15–3.5)

Most frequent underlying diseases	None (*n* = 6) MS (*n* = 4) Crohn's disease (*n* = 2) Arthritis (*n* = 2)	MS (*n* = 4) Eczema (*n* = 2) RA (*n* = 2)	MS (*n* = 2) RA (*n* = 2) Spondylitis (*n* = 2)	Psoriatic arthritis (*n* = 2)	RA and JIA (*n* = 4) Polycythemia vera (*n* = 2)	None (*n* = 3)

Frequent immunophenotyping for diagnosis	CD30+ (*n* = 13, 76.5%) CD3+ (*n* = 10, 59%) CD4+ (*n* = 8, 47%)	CD30+ (*n* = 13, 100%) CD3+ (*n* = 8, 61.5%) ALK− (*n* = 7, 54%)	CD3+ (*n* = 6, 54.5%) CD4 (*n* = 5, 45.5%) TCRγ (*n* = 3, 27%)	CD4+ (*n* = 6, 75%) CD3+ (*n* = 3, 37.5%) TCRβ+ (*n* = 3, 37.5%) TCRγ+ (*n* = 2, 25%)	CD8+ (*n* = 7, 87.5%) CD3+ (*n* = 7, 87.5%) TCRβF1 (*n* = 4, 50%)	CD4+ (*n* = 4, 80%) CD3+ (*n* = 3, 60%) PD1+ (*n* = 3, 60%)

*Note:* SPTCL: subcutaneous panniculitis-like T-cell lymphoma, PCSM-TCL: primary cutaneous CD4+ small/medium-sized T-cell lymphoma.

Abbreviations: JIA, juvenile idiopathic arthritis; LyP, lymphomatoid papulosis; MF, mycosis fungoides; MS, multiple sclerosis; MTX, methotrexate; PC-ALCL, primary cutaneous anaplastic large-cell lymphoma; RA, rheumatoid arthritis; SS, Sézary syndrome.

**Table 6:** Naranjo adverse drug reaction probability scale of the most frequent medications.

Drug/vaccine	Number of cases	Naranjo ADR score (mean ± SE, median (IQR))	Naranjo ADR score (mean ± SD)	Range	Probability
Cyclosporine	4	5.2 ± 0.2, 5 (5–5.75)	5.2 ± 0.5	5–6	Probable
Adalimumab	4	3.5 ± 0.2, 3.5 (3–4)	4.2 ± 0.8	3–5	Possible
Etanercept	5	4.2 ± 0.3, 4 (3.5–5)	3.5 ± 0.5	3-4	Possible
Infliximab	6	4.1 ± 0.6, 4.5 (2.75–5.25)	4.1 ± 1.4	2–6	Possible
Methotrexate	7	4 ± 0.4, 4 (3–5)	4 ± 1.15	3–6	Possible
Fingolimod	8	3.6 ± 0.2, 4 (3.25–4)	3.6 ± 0.7	2–4	Possible
Pfizer–BioNTech vaccine	11	5.5 ± 0.5, 5 (5–7)	5.5 ± 1.6	2–8	Probable

## Data Availability

Data sharing is not applicable to this article as no new data were created or analyzed in this study.

## Supporting Information

Supporting File 1 shows the keywords and search queries we used in this systematic review.

## References

[1] Hristov A. C., Tejasvi T., Wilcox R. A. (2023). Cutaneous T‐cell Lymphomas: 2023 Update on Diagnosis, Risk‐Stratification, and Management. *American Journal of Hematology*.

[2] Agar N. S., Wedgeworth E., Crichton S (2010). Survival Outcomes and Prognostic Factors in Mycosis fungoides/Sézary Syndrome: Validation of the Revised International Society for Cutaneous Lymphomas/European Organisation for Research and Treatment of Cancer Staging Proposal. *Journal of Clinical Oncology*.

[3] Nasimi M., Kamyab K., Aghahi T., Fahim S., Ghandi N. (2020). Childhood Mycosis Fungoides: A Clinicopathologic Study of 30 Cases From Iran. *Australasian Journal of Dermatology*.

[4] Willemze R., Cerroni L., Kempf W (2019). The 2018 Update of the WHO-EORTC Classification for Primary Cutaneous Lymphomas. *Blood*.

[5] Nourmohammadpour P., Nasimi M., Aryanian Z., Goodarzi A., Jahazi R., Etesami I. (2023). Characteristics Associated With Quality of Life in the Early Stages of Mycosis Fungoides. *Caspian Journal of Internal Medicine*.

[6] Morgenroth S., Roggo A., Pawlik L., Dummer R., Ramelyte E. (2023). What Is New in Cutaneous T Cell Lymphoma?. *Current Oncology Reports*.

[7] Stadler R., Stranzenbach R. (2018). Molecular Pathogenesis of Cutaneous Lymphomas. *Experimental Dermatology*.

[8] Dequidt L., Franck N., Sanchez‐Pena P (2019). Cutaneous Lymphomas Appearing During Treatment WITH Biologics: 44 Cases FROM the French Study Group on Cutaneous Lymphomas and French Pharmacovigilance Database. *British Journal of Dermatology*.

[9] Vahabi S. M., Bahramian S., Esmaeili F (2024). JAK Inhibitors in Cutaneous T-Cell Lymphoma: Friend or Foe? A Systematic Review of the Published Literature. *Cancers*.

[10] Lavin L., Dusza S., Geller S. (2024). Cutaneous T-Cell Lymphoma Following Dupilumab Use-A Real-World Pharmacovigilance Study of the FDA Adverse Event Reporting System (FAERS). *Journal of Investigative Dermatology*.

[11] Stuver R., Dusza S., Epstein-Peterson Z. D (2023). Cutaneous T-Cell Lymphoma and Dupilumab Use: A Retrospective Matched Cohort Study of Clinical Characteristics and Treatment Outcomes. *Blood*.

[12] Zheng Y. J., Lee A., Pincus L., Ho W., Vujic M., Ortiz-Urda S. (2018). Cutaneous CD56+ T-Cell Lymphoma Developing During Pembrolizumab Treatment for Metastatic Melanoma. *JAAD Case Reports*.

[13] Tajima S., Takanashi Y., Koda K., Fukayama M. (2015). Methotrexate‐Associated Lymphoproliferative Disorder Presenting as Extranodal NK/T‐Cell Lymphoma Arising in the Lungs. *Pathology International*.

[14] Revenga‐Porcel L., Peñate Y., Granados‐Pacheco F. (2023). Anaplastic Large Cell Lymphoma at the SARS‐CoV2 Vaccine Injection Site. *Journal of the European Academy of Dermatology and Venereology: JEADV*.

[15] Page M. J., McKenzie J. E., Bossuyt P. M. (2021). The PRISMA 2020 Statement: An Updated Guideline for Reporting Systematic Reviews. *Bmj*.

[16] Park A., Wong L., Lang A., Kraus C., Anderson N., Elsensohn A. (2023). Cutaneous T‐Cell Lymphoma Following Dupilumab Use: A Systematic Review. *International Journal of Dermatology*.

[17] Naranjo C. A., Busto U., Sellers E. M. (1981). A Method for Estimating the Probability of Adverse Drug Reactions. *Clinical Pharmacology & Therapeutics*.

[18] Doyle M., Anderson S., Cerrezuela C., Gabrail N. (1994). Sézary Syndrome Associated With Phenytoin Therapy. *Acta Haematologica*.

[19] Riyaz N., Nair L. (1999). Atenolol-Induced Psoriasiform Photodermatitis Evolving Into Sezary Syndrome-A Case Report. *Indian Journal of Dermatology, Venereology and Leprology*.

[20] Di Lernia V., Viglio A., Cattania M., Paulli M. (2001). Carbamazepine-induced, CD30+, Primary, Cutaneous, Anaplastic Large-Cell Lymphoma. *Archives of Dermatology*.

[21] Kirbya B., Owena C. M., Blewittb R. W., Yatesa V. M. (2002). Cutaneous T-Cell Lymphoma Developing in a Patient on Cyclosporin Therapy. *Journal of the American Academy of Dermatology*.

[22] Corazza M., Zampino M. R., Montanari A., Altieri E., Virgili A. (2003). Primary Cutaneous CD30+ Large T-Cell Lymphoma in a Patient With Psoriasis Treated With Cyclosporine. *Dermatology*.

[23] Laube S., Stephens M., Smith A., Whittaker S., Tan B. (2005). Lymphomatoid Papulosis in a Patient With Atopic Eczema on Long‐Term Ciclosporin Therapy. *British Journal of Dermatology*.

[24] Cox N., Madan V., Popple A., Angus B. (2008). CD30‐Positive Lymphoproliferative Disorder With Lesions at Depot Injection Sites, Associated With Mycosis Fungoides and Prostatic Carcinoma. *Clinical and Experimental Dermatology*.

[25] Madray M. M., Greene J. F., Butler D. F. (2008). Glatiramer Acetate–Associated, CD30+, Primary, Cutaneous, Anaplastic Large-Cell Lymphoma. *Archives of Neurology*.

[26] Parker S. R., Solomon A. R., Lane J. E. (2008). A Report of Epstein-Barr Virus–Positive Primary Cutaneous Natural Killer–/t-Cell Lymphoma. *Journal of the American Academy of Dermatology*.

[27] Nemoto Y., Taniguchi A., Kamioka M. (2010). Epstein–Barr Virus-Infected Subcutaneous Panniculitis-Like T-Cell Lymphoma Associated With Methotrexate Treatment. *International Journal of Hematology*.

[28] Alloo A., DeSimone J. A., Kupper T. S. (2012). Mycosis Fungoides Presenting at the Site of a Transdermal Estradiol Patch. *Journal of the American Academy of Dermatology*.

[29] Peck J. R., Frank M. P., Peck L. R. (2013). Was Treatment the Trigger? Mycosis Fungoides. *The American Journal of Medicine*.

[30] Gill K., Ariyan C., Wang X., Brady M. S., Pulitzer M. (2014). CD30‐Positive Lymphoproliferative Disorders Arising After Regional Therapy for Recurrent Melanoma: A Report of Two Cases and Analysis of CD30 Expression. *Journal of Surgical Oncology*.

[31] Samaraweera A. P., Cohen S. N., Akay E. M., Evangelou N. (2016). Lymphomatoid Papulosis: a Cutaneous Lymphoproliferative Disorder in a Patient on Fingolimod for Multiple Sclerosis. *Multiple Sclerosis Journal*.

[32] Claudino W. M., Gibson B., Tse W., Krem M., Grewal J. (2016). Methotrexate-associated Primary Cutaneous CD30-Positive Cutaneous T-Cell Lymphoproliferative Disorder: A Case Illustration and a Brief Review. *American journal of blood research*.

[33] Cohen V., Saber M., Provost N., Friedmann D. (2016). Lymphomatoid Papulosis and Fingolimod—A New Connection? Multiple Sclerosis. *Multiple Sclerosis Journal*.

[34] Papathemeli D., Gräfe R., Hildebrandt U., Zettl U. K., Ulrich J. (2016). Development of a Primary Cutaneous CD30 (+) Anaplastic Large-Cell T-Cell Lymphoma during Treatment of Multiple Sclerosis With Fingolimod. *Multiple Sclerosis Journal*.

[35] Matoula T., Nikolaou V., Marinos L (2016). Lymphomatoid Papulosis Type D in a Fingolimod-Treated Multiple Sclerosis Patient. *Multiple Sclerosis Journal*.

[36] Nitsan Z., Kucuk N., Appel S., Tichmanovich N., Osherov M., Milo R. (2018). Mycosis Fungoides–A Cutaneous Lymphoproliferative Disorder in a Patient Treated With Fingolimod for Multiple Sclerosis. *Journal of Clinical Neuroscience*.

[37] Manouchehri N., Mirmosayyeb O., Badihian S., Shaygannejad V. (2018). Cutaneous Anaplastic Large Cell Lymphoma in a Multiple Sclerosis Patient Receiving Fingolimod. *Multiple Sclerosis and Related Disorders*.

[38] Froehlich A., Schmidt S., Landsberg J., Bieber T., Wenzel J. (2018). Spontaneous Regression of Tumor-Stage Cutaneous T-Cell Lymphoma in a Multiple Sclerosis Patient after Discontinuing Fingolimod. *Multiple Sclerosis Journal*.

[39] Connolly A., Grandi V., Stefanato C., Palmer R., Weir A., Whittaker S. (2018). Primary Cutaneous CD30+ Anaplastic Large‐Cell Lymphoma Associated With Fingolimod. *British Journal of Dermatology*.

[40] Gambichler T., Patsinakidis N., Susok L., Segert M., Doerler M. (2020). Primary Cutaneous CD30+ Anaplastic Large T Cell Lymphoma in a Patient Treated With Cyclosporine for Actinic Reticuloid. *Case Reports in Dermatological Medicine*.

[41] Omori I., Kawanabe R., Hashimoto Y. (2021). Cutaneous Methotrexate-Related T-Cell Lymphoproliferative Disorder With CD4, CD30, CD56, EBV-Positive Tumor Cell Infiltration: A Case Illustration and a Brief Review. *American Journal of Blood Research*.

[42] Matsuoka A., Fujii K., Higashi Y. (2022). Subcutaneous Panniculitis-Like T-Cell Lymphoma Associated With Methotrexate Treatment. *The Journal of Dermatology*.

[43] Sugimoto A., Fujimoto M., Fujii H. (2022). Fatal Case of Methotrexate‐Associated Primary Cutaneous Extranodal NK/T‐Cell Lymphoma of Gamma Delta Phenotype. *Histopathology*.

[44] Masuda Y., Imura K., Sano Y., Yagi H. (2024). Methotrexate-Associated Lymphoproliferative Disorder Presenting as Primary Cutaneous Anaplastic Large Cell Lymphoma With Generalized Skin Lesions. *The Journal of Dermatology*.

[45] Magro C. M., Kalomeris T., Shreve C. R., Geyer J. T., Patel S. S. (2024). Subcutaneous Panniculitic-Like T-Cell Lymphoma Localized to a Site of Peginterferon Alfa-2a Administration. *Leukemia and Lymphoma*.

[46] Mahe E., Descamps V., Grossin M., Fraitag S., Crickx B. (2003). CD30+ T‐Cell Lymphoma in a Patient With Psoriasis Treated With Ciclosporin and Infliximab. *British Journal of Dermatology*.

[47] Adams A. E., Zwicker J., Curiel C. (2004). Aggressive Cutaneous T-Cell Lymphomas After TNFα Blockade. *Journal of the American Academy of Dermatology*.

[48] Dalle S., Balme B., Berger F., Hayette S., Thomas L. (2005). Mycosis Fungoides‐Associated Follicular Mucinosis Under Adalimumab. *British Journal of Dermatology*.

[49] Dauendorffer J., Rivet J., Allard A., Bachelez H. (2007). Sezary Syndrome in a Patient Receiving Infliximab for Ankylosing Spondylitis. *British Journal of Dermatology*.

[50] Koens L., Senff N. J., Vermeer M. H., Ronday H. K., Willemze R., Jansen P. M. (2009). Cutaneous Gamma/delta T-Cell Lymphoma during Treatment With Etanercept for Rheumatoid Arthritis. *Acta Dermato-Venereologica*.

[51] Lourari S., Prey S., Livideanu C. (2009). Cutaneous T‐Cell Lymphoma Following Treatment of Rheumatoid Arthritis With Tumour Necrosis Factor‐α Blocking Agents: Two Cases. *Journal of the European Academy of Dermatology and Venereology*.

[52] Michot C., Costes V., Gerard‐Dran D., Guillot B., Combes B., Dereure O. (2009). Subcutaneous Panniculitis‐Like T‐Cell Lymphoma in a Patient Receiving Etanercept for Rheumatoid Arthritis. *British Journal of Dermatology*.

[53] Outlaw W., Fleischer A., Bloomfeld R. (2009). Lymphomatoid Papulosis in a Patient With Crohn’s Disease Treated With Infliximab. *Inflammatory Bowel Diseases*.

[54] Sluzevich J. C., Hall M. R., Roy V. (2012). Subcutaneous Panniculitis–Like T-Cell Lymphoma After Rituximab. *Journal of the American Academy of Dermatology*.

[55] Bittencourt A. L., Oliveira P. D., Bittencourt V. G., Carvalho E. M., Farre L. (2013). Adult T-Cell Leukemia/Lymphoma Triggered by Adalimumab. *Journal of Clinical Virology*.

[56] Jacks S. M., Taylor B. R., Rogers R. P., Ralston J. S., Metcalf J. S., Lazarchick J. (2014). Rapid Deterioration in a Patient With Primary Aggressive Cutaneous Epidermotropic CD8+ Cytotoxic T-Cell (‘Berti’) Lymphoma After Administration of Adalimumab. *Journal of the American Academy of Dermatology*.

[57] Ma H., Qiu S., Lu R., Feng P., Lu C. (2016). Methotrexate and Etanercept-Induced Primary Cutaneous CD4 Positive Small/Medium-Sized Pleomorphic T-Cell Lymphoma. *Anais Brasileiros de Dermatologia*.

[58] Nakamura H., Sugai T., Kato M., Hatanaka K. C., Atsumi T. (2017). Subcutaneous Panniculitis-Like T-Cell Lymphoma With Haemophagocytic Syndrome During Tocilizumab Therapy for Juvenile Idiopathic Arthritis. *Clinical & Experimental Rheumatology*.

[59] Szakonyi J., Medvecz M., Wikonkál N. (2019). Lymphoproliferative Diseases Among Patients Treated With Biologicals: A Case Study and Single Center Survey. *European Journal of Cancer*.

[60] Cortonesi G., Trovato E., Cinotti E., Gentileschi S., Frediani B., Rubegni P. (2020). Sézary Syndrome During Anti‐IL17 Treatment. *Dermatologic Therapy*.

[61] Shin J., Ho Lee D., Lee W.-J., Park C.-S. (2020). Mycosis Fungoides Development After Combined Immune Checkpoint Blockade Therapy in a Patient With Malignant Melanoma: A Case Report. *Melanoma Research*.

[62] Yasuda T., Takagi T., Asai J. (2021). Mycosis Fungoides in a Patient With Ulcerative Colitis on Anti-Tumor Necrosis Factor-Alpha Therapy. *Clinical Journal of Gastroenterology*.

[63] Barré M., Amatore F., Avenin M., Gorvel L., Olive D., Delaporte E. (2022). Occurrence of Sézary Syndrome Following the Initiation of Anti-IL-5 Treatment. *European Journal of Dermatology: EJD*.

[64] Hida Y., Kageji R., Bekku H., Watanabe S., Seike T., Yamashita M. (2022). Cutaneous T-Cell Lymphoma Developing During Nivolumab Treatment for Metastatic Melanoma. *Dermatology Online Journal*.

[65] Avallone G., Maronese C. A., Conforti C. (2023). Real‐World Data on Primary Cutaneous Lymphoproliferative Disorders Following SARS‐CoV‐2 Vaccination: A Multicentre Experience From Tertiary Referral Hospitals. *Journal of the European Academy of Dermatology and Venereology: JEADV*.

[66] Hooper M. J., Veon F. L., LeWitt T. M. (2022). Cutaneous T-Cell–Rich Lymphoid Infiltrates After SARS-CoV-2 Vaccination. *JAMA dermatology*.

[67] Koumaki D., Marinos L., Nikolaou V. (2022). Lymphomatoid Papulosis (LyP) After AZD1222 and BNT162b2 COVID‐19 Vaccines. *International Journal of Dermatology*.

[68] Kreher M. A., Ahn J., Werbel T., Motaparthi K. (2022). Subcutaneous Panniculitis-Like T-Cell Lymphoma After COVID-19 Vaccination. *JAAD Case Reports*.

[69] Ceravalls J., Arandes-Marcocci J., Pérez-Muñoz N., Fernández-Figueras M., Amores-Martin E. (2023). Painful Palmoplantar Lesions Following Vaccination: Answer. *The American Journal of Dermatopathology*.

[70] Gordon E. R., Kwinta B. D., Schreidah C. M. (2024). Cutaneous Lymphoproliferative Disorders After COVID-19 Vaccination: Clinical Presentation, Histopathology, and Outcomes. *Leukemia and Lymphoma*.

[71] Hobayan C. G., Chung C. G. (2023). Indolent Cutaneous Lymphoma With Gamma/Delta Expression After COVID-19 Vaccination. *JAAD Case Reports*.

[72] Garrido M. C., Riveiro-Falkenbach E., Ruano Y., Ortiz P., Rodriguez-Peralto J. L. (2015). Primary Cutaneous Small/Medium CD4+ T-Cell Lymphoma Occurring During Treatment With Vemurafenib for Advanced Melanoma. *The American Journal of Dermatopathology*.

[73] Iinuma S., Hayashi K., Noguchi A., Ishida-Yamamoto A. (2022). Lymphomatoid Papulosis During Upadacitinib Treatment for Rheumatoid Arthritis. *European Journal of Dermatology: EJD*.

[74] Knapp C., Steele E., Mengden-Koon S., Williams T., Fett N. (2022). A Case of Tofacitinib-Induced Lymphomatoid Papulosis With Ocular Involvement. *The American Journal of Dermatopathology*.

[75] Saito K., Shimauchi T., Kageyama R. (2023). A Case of Sézary Syndrome in a Patient During Treatment With Baricitinib for Seronegative Rheumatoid Arthritis. *Clinical and Experimental Dermatology*.

[76] Moon I. J., Won C. H., Chang S. E. (2024). Prevalence, Clinical Features, and Survival Outcome Trends of 627 Patients With Primary Cutaneous Lymphoma over 29 years: a Retrospective Review From Single Tertiary Center in Korea. *Scientific Reports*.

[77] Martinez‐Cabriales S., Walsh S., Sade S., Shear N. (2020). Lymphomatoid Papulosis: An Update and Review. *Journal of the European Academy of Dermatology and Venereology*.

[78] Nikolaenko L., Zain J., Rosen S. T., Querfeld C. (2019). CD30-Positive Lymphoproliferative Disorders. *T-cell and NK-Cell Lymphomas: From Biology to Novel Therapies*.

[79] Nowicka D., Mertowska P., Mertowski S. (2022). Etiopathogenesis, Diagnosis, and Treatment Strategies for Lymphomatoid Papulosis With Particular Emphasis on the Role of the Immune System. *Cells*.

[80] Ortiz-Hidalgo C., Pina-Oviedo S. (2023). Primary Cutaneous Anaplastic Large Cell Lymphoma—A Review of Clinical, Morphological, Immunohistochemical, and Molecular Features. *Cancers*.

[81] Di Raimondo C., Parekh V., Song J. Y. (2020). Primary Cutaneous CD30+ Lymphoproliferative Disorders: A Comprehensive Review. *Current Hematologic Malignancy Reports*.

[82] García-Díaz N., Piris M. Á, Ortiz-Romero P. L., Vaqué J. P. (2021). Mycosis Fungoides and Sézary Syndrome: An Integrative Review of the Pathophysiology, Molecular Drivers, and Targeted Therapy. *Cancers*.

[83] Schaefer L., Comfere N., Sokumbi O. (2023). Development of Cutaneous T-Cell Lymphoma Following Biologic Treatment: A Systematic Review. *American Journal of Clinical Dermatology*.

[84] Martinez-Escala M. E., Posligua A. L., Wickless H. (2018). Progression of Undiagnosed Cutaneous Lymphoma After Anti–Tumor Necrosis Factor-Alpha Therapy. *Journal of the American Academy of Dermatology*.

[85] Moustou A.-E., Matekovits A., Dessinioti C., Antoniou C., Sfikakis P. P., Stratigos A. J. (2009). Cutaneous Side Effects of Anti–Tumor Necrosis Factor Biologic Therapy: A Clinical Review. *Journal of the American Academy of Dermatology*.

[86] Kappos L., O’Connor P., Radue E.-W. (2015). Long-Term Effects of Fingolimod in Multiple Sclerosis: The Randomized FREEDOMS Extension Trial. *Neurology*.

[87] Lorvik K. B., Bogen B., Corthay A. (2012). Fingolimod Blocks Immunosurveillance of Myeloma and B-Cell Lymphoma Resulting in Cancer Development in Mice. *Blood*.

[88] Muellenhoff M. W., Koo J. Y. (2012). Cyclosporine and Skin Cancer: An International Dermatologic Perspective Over 25 Years of Experience. A Comprehensive Review and Pursuit to Define Safe Use of Cyclosporine in Dermatology. *Journal of Dermatological Treatment*.

[89] Penn I. (1987). Cancers Following Cyclosporins Therapy. *Transplantation*.

[90] Opelz G., Döhler B. (2004). Lymphomas After Solid Organ Transplantation: A Collaborative Transplant Study Report. *American Journal of Transplantation*.

[91] Oh Y., Stoll J. R., Moskowitz A. (2021). Primary Cutaneous T-Cell Lymphomas Other Than Mycosis Fungoides and Sézary Syndrome. Part II: Prognosis and Management. *Journal of the American Academy of Dermatology*.

[92] Lee W. J., Moon I. J., Lee S. H. (2016). Cutaneous Anaplastic Large-Cell Lymphoma (ALCL): A Comparative Clinical Feature and Survival Outcome Analysis of 52 Cases According to Primary Tumor Site. *Journal of the American Academy of Dermatology*.

[93] Latzka J., Assaf C., Bagot M. (2023). EORTC Consensus Recommendations for the Treatment of Mycosis Fungoides/Sézary Syndrome–Update 2023. *European Journal of Cancer*.

