# Supplementary material for: Drug- and Vaccine-Induced Cutaneous T-Cell Lymphoma: A Systematic Review of the Literature
Source: J Skin Cancer. 2025 Feb 27;2025:3103865. doi: 10.1155/jskc/3103865 (PMC11986929; doi:10.1155/jskc/3103865)
Supplement: Supporting Information — Additional supporting information can be found online in the Supporting Information section. [file 3103865.f1.docx]

Pubmed: (2363 results)

(”Lymphoma, T-Cell, Cutaneous”[Mesh] OR “Lymphoma, Primary Cutaneous Anaplastic Large Cell”[Mesh] OR “Lymphomatoid Papulosis”[Mesh] OR “Mycosis Fungoides”[Mesh] OR “Pagetoid Reticulosis”[Mesh] OR “Sezary Syndrome”[Mesh] OR “Cutaneous T-Cell Lymphoma”[tiab] OR “Cutaneous T Cell Lymphoma”[tiab] OR “Cutaneous T-Cell Lymphomas”[tiab] OR “Granulomatous Slack Skin”[tiab] OR “Primary Cutaneous CD30-positive Large T-Cell Lymphoma”[tiab] OR “Primary Cutaneous CD30 positive Large T Cell Lymphoma”[tiab] OR “Primary Cutaneous Anaplastic Large Cell Lymphoma”[tiab] OR “Lymphomatoid Papuloses”[tiab] OR “Mycosis Fungoides”[tiab] OR “Pagetoid Reticulosis”[tiab] OR “Pagetoid Reticuloses”[tiab] OR “Woringer-Kolopp Disease”[tiab] OR “Woringer Kolopp Disease”[tiab] OR “Ketron-Goodman Disease”[tiab] OR “Ketron Goodman Disease”[tiab] OR “Sezary Syndrome”[tiab] OR “Sezary's Lymphoma”[tiab] OR “Sezary Lymphoma”[tiab] OR “Sezarys Lymphoma”[tiab] OR “Sezary Erythroderma”[tiab] OR “ALCL of the skin”[tiab] OR “C-ALCL”[tiab] OR “Ki-1 lymphoma of the skin”[tiab] OR “primary anaplastic large cell lymphoma of the skin”[tiab] OR “primary cutaneous anaplastic large T-cell lymphoma”[tiab] OR “primary cutaneous CD30 positive anaplastic large cell lymphoma”[tiab] OR “primary cutaneous CD30 positive anaplastic large T-cell lymphoma”[tiab] OR “primary cutaneous CD30+ ALCL”[tiab] OR “primary cutaneous CD30+ anaplastic large cell lymphoma”[tiab] OR “primary cutaneous CD30+ anaplastic large T-cell lymphoma”[tiab] OR “primary cutaneous CD30+ large T-cell lymphoma”[tiab] OR “primary cutaneous CD30-positive large T-cell lymphoma”[tiab] OR “regressing atypical histiocytosis”[tiab] OR “regressive atypical histiocytosis”[tiab] OR “primary cutaneous anaplastic large cell lymphoma”[tiab] OR “lymphogranulomatosis X”[tiab] OR “angioimmunoblastic T cell lymphoma”[tiab] OR “cutaneous T-cell lymphoma”[tiab] OR “cutaneous T-cell non-Hodgkin lymphoma”[tiab] OR “cutaneous T-cell non-Hodgkin's lymphoma”[tiab] OR “cutaneous TCL”[tiab] OR “primary cutaneous T cell lymphoma”[tiab] OR “T-cell cutaneous lymphoma”[tiab] OR “cutaneous T cell lymphoma”[tiab] OR “aggressive cutaneous T-cell lymphoma”[tiab] OR “aggressive primary cutaneous T-cell lymphoma”[tiab] OR “aggressive primary cutaneous T cell lymphoma”[tiab] OR “CGD-TCL”[tiab] OR “CGDTCL”[tiab] OR “cutaneous g and d T cell lymphoma”[tiab] OR “cutaneous g/d T cell lymphoma”[tiab] OR “cutaneous gamma-delta TCL”[tiab] OR “cutaneous gamma/delta T cell lymphoma”[tiab] OR “cutaneous gammadelta T cell lymphoma”[tiab] OR “cutaneous gd T cell lymphoma”[tiab] OR “cutaneous gd-TCL”[tiab] OR “cutaneous GDTCL”[tiab] OR “cutaneous T cell lymphoma gamma delta subtype”[tiab] OR “gamma/delta T-cell lymphoma of the skin”[tiab] OR “PCGD-TCL”[tiab] OR “PCGDTCL”[tiab] OR “primary cutaneous gamma delta T cell lymphoma”[tiab] OR “primary cutaneous gammadelta T cell lymphoma”[tiab] OR “cutaneous gamma delta T cell lymphoma”[tiab] OR “aggressive epidermotropic CD8+ T-cell lymphoma”[tiab] OR “aggressive epidermotropic CD8-positive T-cell lymphoma”[tiab] OR “Berti lymphoma”[tiab] OR “Berti's lymphoma”[tiab] OR “PCAETCL”[tiab] OR “primary cutaneous aggressive epidermotropic CD8 T-cell lymphoma”[tiab] OR “primary cutaneous aggressive epidermotropic CD8+ T-cell lymphoma”[tiab] OR “primary cutaneous aggressive epidermotropic CD8+ T cell lymphoma”[tiab] OR “cutaneous T-Cell lymphoma/Sezary syndrome”[tiab] OR “Sezary disease”[tiab] OR “Sezary erythroderma”[tiab] OR “Sezary lymphoma”[tiab] OR “Sezary's disease”[tiab] OR “Sezary's reticulosis”[tiab] OR “Sezary's syndrome”[tiab] OR “Sezarys syndrome”[tiab] OR “syndrome sezary”[tiab] OR “Sezary syndrome”[tiab] OR “angiocentric cutaneous T-cell lymphoma of childhood”[tiab] OR “HV-like lymphoma”[tiab] OR “hydroa vacciniforme-like lymphoproliferative disease”[tiab] OR “hydroa vacciniforme-like lymphoproliferative disorder”[tiab] OR “hydroa-like cutaneous T-cell lymphoma”[tiab] OR “hydroa-like lymphoma”[tiab] OR “hydroa vacciniforme-like lymphoma”[tiab] OR “indolent cutaneous T-cell lymphoma”[tiab] OR “indolent primary cutaneous T-cell lymphoma”[tiab] OR “indolent primary cutaneous T cell lymphoma”[tiab] OR “cutaneous T-cell lymphoma/mycosis fungoides”[tiab] OR “granuloma fungoides”[tiab] OR “mycoses fungoides”[tiab] OR “mycosis fungoides lymphoma”[tiab] OR “mycosis fungoides”[tiab] OR “Alibert Bazin disease”[tiab] OR “Alibert-Bazin syndrome”[tiab] OR “Alibert-Bazin type mycosis fungoides”[tiab] OR “classic MF”[tiab] OR “classical MF”[tiab] OR “classical mycosis fungoides”[tiab] OR “disease, Alibert Bazin”[tiab] OR “classic mycosis fungoides”[tiab] OR “folliculotropic MF”[tiab] OR “mycosis fungoides-associated follicular mucinosis”[tiab] OR “folliculotropic mycosis fungoides”[tiab] OR “granulomatous MF”[tiab] OR “granulomatous mycosis fungoides”[tiab] OR “granulomatous slack skin”[tiab] OR “disseminated pagetoid reticulosis”[tiab] OR “epidermotropic reticulosis”[tiab] OR “generalized pagetoid reticulosis”[tiab] OR “Ketron-Goodman”[tiab] OR “Ketron-Goodman disease”[tiab] OR “localized pagetoid reticulosis”[tiab] OR “Woringer-Kolopp disease”[tiab] OR “pagetoid reticulosis”[tiab] OR “CD30 LPD”[tiab] OR “CD30+ LPD”[tiab] OR “CD30-positive LPD”[tiab] OR “cutaneous CD30+ T-cell lymphoproliferative disease”[tiab] OR “cutaneous CD30+ T-cell lymphoproliferative diseases”[tiab] OR “cutaneous CD30+ T-cell lymphoproliferative disorder”[tiab] OR “cutaneous CD30+ T-cell lymphoproliferative disorders”[tiab] OR “primary cutaneous CD30+ T-cell lymphoproliferative disease”[tiab] OR “primary cutaneous CD30+ T-cell lymphoproliferative diseases”[tiab] OR “primary cutaneous CD30+ T-cell lymphoproliferative disorder”[tiab] OR “primary cutaneous CD30+ T-cell lymphoproliferative disorders”[tiab] OR “primary cutaneous CD30+ T cell lymphoproliferative disorder”[tiab] OR “LyP”[tiab] OR “lymphomatoid papulosis”[tiab] OR “ALCL of the skin”[tiab] OR “C-ALCL”[tiab] OR “Ki-1 lymphoma of the skin”[tiab] OR “primary anaplastic large cell lymphoma of the skin”[tiab] OR “primary cutaneous anaplastic large T-cell lymphoma”[tiab] OR “primary cutaneous CD30 positive anaplastic large cell lymphoma”[tiab] OR “primary cutaneous CD30 positive anaplastic large T-cell lymphoma”[tiab] OR “primary cutaneous CD30+ ALCL”[tiab] OR “primary cutaneous CD30+ anaplastic large cell lymphoma”[tiab] OR “primary cutaneous CD30+ anaplastic large T-cell lymphoma”[tiab] OR “primary cutaneous CD30+ large T-cell lymphoma”[tiab] OR “primary cutaneous CD30-positive large T-cell lymphoma”[tiab] OR “regressing atypical histiocytosis”[tiab] OR “regressive atypical histiocytosis”[tiab] OR “primary cutaneous anaplastic large cell lymphoma”[tiab] OR “SP-TCL”[tiab] OR “SPTCL”[tiab] OR “subcutaneous panniculitic T-cell lymphoma”[tiab] OR “subcutaneous panniculitis like T cell lymphoma”[tiab] OR “subcutaneous panniculitis T-cell lymphoma”[tiab] OR “subcutaneous panniculitis-like TCL”[tiab] OR “subcutaneous T cell lymphoma”[tiab]) AND (“Drug-Related Side Effects and Adverse Reactions”[Mesh] OR “drug”[tiab] OR “drug-induced”[tiab] OR “induced”[tiab] OR “induction”[tiab] OR “trigger”[tiab] OR “triggered”[tiab] OR “Jak inhibitor”[tiab] OR “Romidepsin”[tiab] OR “afatinib synergism”[tiab] OR “Thiazides”[tiab] OR “topical calcineurin inhibitors”[tiab] OR “tacrolimus”[tiab] OR “pimecrolimus”[tiab] OR “Methotrexate”[tiab])

Embase: (6483 results)

‘primary cutaneous anaplastic large cell lymphoma’/exp OR ‘cutaneous T cell lymphoma’/exp OR ‘cutaneous gamma delta T cell lymphoma’/exp OR ‘primary cutaneous aggressive epidermotropic CD8+ T cell lymphoma’/exp OR ‘Sezary syndrome’/exp OR ‘hydroa vacciniforme-like lymphoma’/exp OR ‘indolent primary cutaneous T cell lymphoma’/exp OR ‘mycosis fungoides’/exp OR ‘classic mycosis fungoides’/exp OR ‘folliculotropic mycosis fungoides’/exp OR ‘granulomatous mycosis fungoides’/exp OR ‘granulomatous slack skin’/exp OR ‘pagetoid reticulosis’/exp OR ‘primary cutaneous CD30+ T cell lymphoproliferative disorder’/exp OR ‘lymphomatoid papulosis’/exp OR ‘primary cutaneous anaplastic large cell lymphoma’/exp OR ‘subcutaneous T cell lymphoma’/exp OR ‘Cutaneous T-Cell Lymphoma’:ab,ti OR ‘Cutaneous T Cell Lymphoma’:ab,ti OR ‘Cutaneous T-Cell Lymphomas’:ab,ti OR ‘Granulomatous Slack Skin’:ab,ti OR ‘Primary Cutaneous CD30-positive Large T-Cell Lymphoma’:ab,ti OR ‘Primary Cutaneous CD30 positive Large T Cell Lymphoma’:ab,ti OR ‘Primary Cutaneous Anaplastic Large Cell Lymphoma’:ab,ti OR ‘Lymphomatoid Papuloses’:ab,ti OR ‘Mycosis Fungoides’:ab,ti OR ‘Pagetoid Reticulosis’:ab,ti OR ‘Pagetoid Reticuloses’:ab,ti OR ‘Woringer-Kolopp Disease’:ab,ti OR ‘Woringer Kolopp Disease’:ab,ti OR ‘Ketron-Goodman Disease’:ab,ti OR ‘Ketron Goodman Disease’:ab,ti OR ‘Sezary Syndrome’:ab,ti OR ‘Sezary Lymphoma’:ab,ti OR ‘Sezarys Lymphoma’:ab,ti OR ‘Sezary Erythroderma’:ab,ti OR ‘ALCL of the skin’:ab,ti OR ‘C-ALCL’:ab,ti OR ‘Ki-1 lymphoma of the skin’:ab,ti OR ‘primary anaplastic large cell lymphoma of the skin’:ab,ti OR ‘primary cutaneous anaplastic large T-cell lymphoma’:ab,ti OR ‘primary cutaneous CD30 positive anaplastic large cell lymphoma’:ab,ti OR ‘primary cutaneous CD30 positive anaplastic large T-cell lymphoma’:ab,ti OR ‘primary cutaneous CD30+ ALCL’:ab,ti OR ‘primary cutaneous CD30+ anaplastic large cell lymphoma’:ab,ti OR ‘primary cutaneous CD30+ anaplastic large T-cell lymphoma’:ab,ti OR ‘primary cutaneous CD30+ large T-cell lymphoma’:ab,ti OR ‘primary cutaneous CD30-positive large T-cell lymphoma’:ab,ti OR ‘regressing atypical histiocytosis’:ab,ti OR ‘regressive atypical histiocytosis’:ab,ti OR ‘primary cutaneous anaplastic large cell lymphoma’:ab,ti OR ‘lymphogranulomatosis X’:ab,ti OR ‘angioimmunoblastic T cell lymphoma’:ab,ti OR ‘cutaneous T-cell lymphoma’:ab,ti OR ‘cutaneous T-cell non-Hodgkin lymphoma’:ab,ti OR ‘cutaneous TCL’:ab,ti OR ‘primary cutaneous T cell lymphoma’:ab,ti OR ‘T-cell cutaneous lymphoma’:ab,ti OR ‘cutaneous T cell lymphoma’:ab,ti OR ‘aggressive cutaneous T-cell lymphoma’:ab,ti OR ‘aggressive primary cutaneous T-cell lymphoma’:ab,ti OR ‘aggressive primary cutaneous T cell lymphoma’:ab,ti OR ‘CGD-TCL’:ab,ti OR ‘CGDTCL’:ab,ti OR ‘cutaneous g and d T cell lymphoma’:ab,ti OR ‘cutaneous g/d T cell lymphoma’:ab,ti OR ‘cutaneous gamma-delta TCL’:ab,ti OR ‘cutaneous gamma/delta T cell lymphoma’:ab,ti OR ‘cutaneous gammadelta T cell lymphoma’:ab,ti OR ‘cutaneous gd T cell lymphoma’:ab,ti OR ‘cutaneous gd-TCL’:ab,ti OR ‘cutaneous GDTCL’:ab,ti OR ‘cutaneous T cell lymphoma gamma delta subtype’:ab,ti OR ‘gamma/delta T-cell lymphoma of the skin’:ab,ti OR ‘PCGD-TCL’:ab,ti OR ‘PCGDTCL’:ab,ti OR ‘primary cutaneous gamma delta T cell lymphoma’:ab,ti OR ‘primary cutaneous gammadelta T cell lymphoma’:ab,ti OR ‘cutaneous gamma delta T cell lymphoma’:ab,ti OR ‘aggressive epidermotropic CD8+ T-cell lymphoma’:ab,ti OR ‘aggressive epidermotropic CD8-positive T-cell lymphoma’:ab,ti OR ‘Berti lymphoma’:ab,ti OR ‘PCAETCL’:ab,ti OR ‘primary cutaneous aggressive epidermotropic CD8 T-cell lymphoma’:ab,ti OR ‘primary cutaneous aggressive epidermotropic CD8+ T-cell lymphoma’:ab,ti OR ‘primary cutaneous aggressive epidermotropic CD8+ T cell lymphoma’:ab,ti OR ‘cutaneous T-Cell lymphoma/Sezary syndrome’:ab,ti OR ‘Sezary disease’:ab,ti OR ‘Sezary erythroderma’:ab,ti OR ‘Sezary lymphoma’:ab,ti OR ‘Sezarys syndrome’:ab,ti OR ‘syndrome sezary’:ab,ti OR ‘Sezary syndrome’:ab,ti OR ‘angiocentric cutaneous T-cell lymphoma of childhood’:ab,ti OR ‘HV-like lymphoma’:ab,ti OR ‘hydroa vacciniforme-like lymphoproliferative disease’:ab,ti OR ‘hydroa vacciniforme-like lymphoproliferative disorder’:ab,ti OR ‘hydroa-like cutaneous T-cell lymphoma’:ab,ti OR ‘hydroa-like lymphoma’:ab,ti OR ‘hydroa vacciniforme-like lymphoma’:ab,ti OR ‘indolent cutaneous T-cell lymphoma’:ab,ti OR ‘indolent primary cutaneous T-cell lymphoma’:ab,ti OR ‘indolent primary cutaneous T cell lymphoma’:ab,ti OR ‘cutaneous T-cell lymphoma/mycosis fungoides’:ab,ti OR ‘granuloma fungoides’:ab,ti OR ‘mycoses fungoides’:ab,ti OR ‘mycosis fungoides lymphoma’:ab,ti OR ‘mycosis fungoides’:ab,ti OR ‘Alibert Bazin disease’:ab,ti OR ‘Alibert-Bazin syndrome’:ab,ti OR ‘Alibert-Bazin type mycosis fungoides’:ab,ti OR ‘classic MF’:ab,ti OR ‘classical MF’:ab,ti OR ‘classical mycosis fungoides’:ab,ti OR ‘disease, Alibert Bazin’:ab,ti OR ‘classic mycosis fungoides’:ab,ti OR ‘folliculotropic MF’:ab,ti OR ‘mycosis fungoides-associated follicular mucinosis’:ab,ti OR ‘folliculotropic mycosis fungoides’:ab,ti OR ‘granulomatous MF’:ab,ti OR ‘granulomatous mycosis fungoides’:ab,ti OR ‘granulomatous slack skin’:ab,ti OR ‘disseminated pagetoid reticulosis’:ab,ti OR ‘epidermotropic reticulosis’:ab,ti OR ‘generalized pagetoid reticulosis’:ab,ti OR ‘Ketron-Goodman’:ab,ti OR ‘Ketron-Goodman disease’:ab,ti OR ‘localized pagetoid reticulosis’:ab,ti OR ‘Woringer-Kolopp disease’:ab,ti OR ‘pagetoid reticulosis’:ab,ti OR ‘CD30 LPD’:ab,ti OR ‘CD30+ LPD’:ab,ti OR ‘CD30-positive LPD’:ab,ti OR ‘cutaneous CD30+ T-cell lymphoproliferative disease’:ab,ti OR ‘cutaneous CD30+ T-cell lymphoproliferative diseases’:ab,ti OR ‘cutaneous CD30+ T-cell lymphoproliferative disorder’:ab,ti OR ‘cutaneous CD30+ T-cell lymphoproliferative disorders’:ab,ti OR ‘primary cutaneous CD30+ T-cell lymphoproliferative disease’:ab,ti OR ‘primary cutaneous CD30+ T-cell lymphoproliferative diseases’:ab,ti OR ‘primary cutaneous CD30+ T-cell lymphoproliferative disorder’:ab,ti OR ‘primary cutaneous CD30+ T-cell lymphoproliferative disorders’:ab,ti OR ‘primary cutaneous CD30+ T cell lymphoproliferative disorder’:ab,ti OR ‘LyP’:ab,ti OR ‘lymphomatoid papulosis’:ab,ti OR ‘ALCL of the skin’:ab,ti OR ‘C-ALCL’:ab,ti OR ‘Ki-1 lymphoma of the skin’:ab,ti OR ‘primary anaplastic large cell lymphoma of the skin’:ab,ti OR ‘primary cutaneous anaplastic large T-cell lymphoma’:ab,ti OR ‘primary cutaneous CD30 positive anaplastic large cell lymphoma’:ab,ti OR ‘primary cutaneous CD30 positive anaplastic large T-cell lymphoma’:ab,ti OR ‘primary cutaneous CD30+ ALCL’:ab,ti OR ‘primary cutaneous CD30+ anaplastic large cell lymphoma’:ab,ti OR ‘primary cutaneous CD30+ anaplastic large T-cell lymphoma’:ab,ti OR ‘primary cutaneous CD30+ large T-cell lymphoma’:ab,ti OR ‘primary cutaneous CD30-positive large T-cell lymphoma’:ab,ti OR ‘regressing atypical histiocytosis’:ab,ti OR ‘regressive atypical histiocytosis’:ab,ti OR ‘primary cutaneous anaplastic large cell lymphoma’:ab,ti OR ‘SP-TCL’:ab,ti OR ‘SPTCL’:ab,ti OR ‘subcutaneous panniculitic T-cell lymphoma’:ab,ti OR ‘subcutaneous panniculitis like T cell lymphoma’:ab,ti OR ‘subcutaneous panniculitis T-cell lymphoma’:ab,ti OR ‘subcutaneous panniculitis-like TCL’:ab,ti OR ‘subcutaneous T cell lymphoma’:ab,ti

AND

‘drug induced disease’/exp OR ‘adverse drug reaction’/exp OR “drug’:ab,ti OR ‘drug-induced’:ab,ti OR ‘induced’:ab,ti OR ‘induction’:ab,ti OR ‘trigger’:ab,ti OR ‘triggered’:ab,ti OR ‘Jak inhibitor’:ab,ti OR ‘Romidepsin’:ab,ti OR ‘afatinib synergism’:ab,ti OR ‘Thiazides’:ab,ti OR ‘topical calcineurin inhibitors’:ab,ti OR ‘tacrolimus’:ab,ti OR ‘pimecrolimus’:ab,ti OR ‘Methotrexate’:ab,ti

Scopus: (9713 results)

“Cutaneous T-Cell Lymphoma” OR “Cutaneous T Cell Lymphoma” OR “Cutaneous T-Cell Lymphomas” OR “Granulomatous Slack Skin” OR “Primary Cutaneous CD30-positive Large T-Cell Lymphoma” OR “Primary Cutaneous CD30 positive Large T Cell Lymphoma” OR “Primary Cutaneous Anaplastic Large Cell Lymphoma” OR “Lymphomatoid Papuloses” OR “Mycosis Fungoides” OR “Pagetoid Reticulosis” OR “Pagetoid Reticuloses” OR “Woringer-Kolopp Disease” OR “Woringer Kolopp Disease” OR “Ketron-Goodman Disease” OR “Ketron Goodman Disease” OR “Sezary Syndrome” OR “Sezary’s Lymphoma” OR “Sezary Lymphoma” OR “Sezary’s Lymphoma” OR “Sezary Erythroderma” OR “ALCL of the skin” OR “C-ALCL” OR “Ki-1 lymphoma of the skin” OR “primary anaplastic large cell lymphoma of the skin” OR “primary cutaneous anaplastic large T-cell lymphoma” OR “primary cutaneous CD30 positive anaplastic large cell lymphoma” OR “primary cutaneous CD30 positive anaplastic large T-cell lymphoma” OR “primary cutaneous CD30+ ALCL” OR “primary cutaneous CD30+ anaplastic large cell lymphoma” OR “primary cutaneous CD30+ anaplastic large T-cell lymphoma” OR “primary cutaneous CD30+ large T-cell lymphoma” OR “primary cutaneous CD30-positive large T-cell lymphoma” OR “regressing atypical histiocytosis” OR “regressive atypical histiocytosis” OR “primary cutaneous anaplastic large cell lymphoma” OR “lymphogranulomatosis X” OR “angioimmunoblastic T cell lymphoma” OR “cutaneous T-cell lymphoma” OR “cutaneous T-cell non-Hodgkin lymphoma” OR “cutaneous T-cell non-Hodgkin’s lymphoma” OR “cutaneous TCL” OR “primary cutaneous T cell lymphoma” OR “T-cell cutaneous lymphoma” OR “cutaneous T cell lymphoma” OR “aggressive cutaneous T-cell lymphoma” OR “aggressive primary cutaneous T-cell lymphoma” OR “aggressive primary cutaneous T cell lymphoma” OR “CGD-TCL” OR “CGDTCL” OR “cutaneous g and d T cell lymphoma” OR “cutaneous g/d T cell lymphoma” OR “cutaneous gamma-delta TCL” OR “cutaneous gamma/delta T cell lymphoma” OR “cutaneous gammadelta T cell lymphoma” OR “cutaneous gd T cell lymphoma” OR “cutaneous gd-TCL” OR “cutaneous GDTCL” OR “cutaneous T cell lymphoma gamma delta subtype” OR “gamma/delta T-cell lymphoma of the skin” OR “PCGD-TCL” OR “PCGDTCL” OR “primary cutaneous gamma delta T cell lymphoma” OR “primary cutaneous gammadelta T cell lymphoma” OR “cutaneous gamma delta T cell lymphoma” OR “aggressive epidermotropic CD8+ T-cell lymphoma” OR “aggressive epidermotropic CD8-positive T-cell lymphoma” OR “Berti lymphoma” OR “Berti’s lymphoma” OR “PCAETCL” OR “primary cutaneous aggressive epidermotropic CD8 T-cell lymphoma” OR “primary cutaneous aggressive epidermotropic CD8+ T-cell lymphoma” OR “primary cutaneous aggressive epidermotropic CD8+ T cell lymphoma” OR “cutaneous T-Cell lymphoma/Sezary syndrome” OR “Sezary disease” OR “Sezary erythroderma” OR “Sezary lymphoma” OR “Sezary’s disease” OR “Sezary’s reticulosis” OR “Sezary’s syndrome” OR “Sezarys syndrome” OR “syndrome sezary” OR “Sezary syndrome” OR “angiocentric cutaneous T-cell lymphoma of childhood” OR “HV-like lymphoma” OR “hydroa vacciniforme-like lymphoproliferative disease” OR “hydroa vacciniforme-like lymphoproliferative disorder” OR “hydroa-like cutaneous T-cell lymphoma” OR “hydroa-like lymphoma” OR “hydroa vacciniforme-like lymphoma” OR “indolent cutaneous T-cell lymphoma” OR “indolent primary cutaneous T-cell lymphoma” OR “indolent primary cutaneous T cell lymphoma” OR “cutaneous T-cell lymphoma/mycosis fungoides” OR “granuloma fungoides” OR “mycoses fungoides” OR “mycosis fungoides lymphoma” OR “mycosis fungoides” OR “Alibert Bazin disease” OR “Alibert-Bazin syndrome” OR “Alibert-Bazin type mycosis fungoides” OR “classic MF” OR “classical MF” OR “classical mycosis fungoides” OR “disease, Alibert Bazin” OR “classic mycosis fungoides” OR “folliculotropic MF” OR “mycosis fungoides-associated follicular mucinosis” OR “folliculotropic mycosis fungoides” OR “granulomatous MF” OR “granulomatous mycosis fungoides” OR “granulomatous slack skin” OR “disseminated pagetoid reticulosis” OR “epidermotropic reticulosis” OR “generalized pagetoid reticulosis” OR “Ketron-Goodman” OR “Ketron-Goodman disease” OR “localized pagetoid reticulosis” OR “Woringer-Kolopp disease” OR “pagetoid reticulosis” OR “CD30 LPD” OR “CD30+ LPD” OR “CD30-positive LPD” OR “cutaneous CD30+ T-cell lymphoproliferative disease” OR “cutaneous CD30+ T-cell lymphoproliferative diseases” OR “cutaneous CD30+ T-cell lymphoproliferative disorder” OR “cutaneous CD30+ T-cell lymphoproliferative disorders” OR “primary cutaneous CD30+ T-cell lymphoproliferative disease” OR “primary cutaneous CD30+ T-cell lymphoproliferative diseases” OR “primary cutaneous CD30+ T-cell lymphoproliferative disorder” OR “primary cutaneous CD30+ T-cell lymphoproliferative disorders” OR “primary cutaneous CD30+ T cell lymphoproliferative disorder” OR “LyP” OR “lymphomatoid papulosis” OR “ALCL of the skin” OR “C-ALCL” OR “Ki-1 lymphoma of the skin” OR “primary anaplastic large cell lymphoma of the skin” OR “primary cutaneous anaplastic large T-cell lymphoma” OR “primary cutaneous CD30 positive anaplastic large cell lymphoma” OR “primary cutaneous CD30 positive anaplastic large T-cell lymphoma” OR “primary cutaneous CD30+ ALCL” OR “primary cutaneous CD30+ anaplastic large cell lymphoma” OR “primary cutaneous CD30+ anaplastic large T-cell lymphoma” OR “primary cutaneous CD30+ large T-cell lymphoma” OR “primary cutaneous CD30-positive large T-cell lymphoma” OR “regressing atypical histiocytosis” OR “regressive atypical histiocytosis” OR “primary cutaneous anaplastic large cell lymphoma” OR “SP-TCL” OR “SPTCL” OR “subcutaneous panniculitic T-cell lymphoma” OR “subcutaneous panniculitis like T cell lymphoma” OR “subcutaneous panniculitis T-cell lymphoma” OR “subcutaneous panniculitis-like TCL” OR “subcutaneous T cell lymphoma”

AND

“adverse drug reaction” OR “drug” OR “drug-induced” OR “induced” OR “induction” OR “trigger” OR “triggered” OR “Jak inhibitor” OR “Romidepsin” OR “afatinib synergism” OR “Thiazides” OR “topical calcineurin inhibitors” OR “tacrolimus” OR “pimecrolimus” OR “Methotrexate”

Web of Science: (2276 results)

“Cutaneous T-Cell Lymphoma” OR “Cutaneous T Cell Lymphoma” OR “Cutaneous T-Cell Lymphomas” OR “Granulomatous Slack Skin” OR “Primary Cutaneous CD30-positive Large T-Cell Lymphoma” OR “Primary Cutaneous CD30 positive Large T Cell Lymphoma” OR “Primary Cutaneous Anaplastic Large Cell Lymphoma” OR “Lymphomatoid Papuloses” OR “Mycosis Fungoides” OR “Pagetoid Reticulosis” OR “Pagetoid Reticuloses” OR “Woringer-Kolopp Disease” OR “Woringer Kolopp Disease” OR “Ketron-Goodman Disease” OR “Ketron Goodman Disease” OR “Sezary Syndrome” OR “Sezary’s Lymphoma” OR “Sezary Lymphoma” OR “Sezary’s Lymphoma” OR “Sezary Erythroderma” OR “ALCL of the skin” OR “C-ALCL” OR “Ki-1 lymphoma of the skin” OR “primary anaplastic large cell lymphoma of the skin” OR “primary cutaneous anaplastic large T-cell lymphoma” OR “primary cutaneous CD30 positive anaplastic large cell lymphoma” OR “primary cutaneous CD30 positive anaplastic large T-cell lymphoma” OR “primary cutaneous CD30+ ALCL” OR “primary cutaneous CD30+ anaplastic large cell lymphoma” OR “primary cutaneous CD30+ anaplastic large T-cell lymphoma” OR “primary cutaneous CD30+ large T-cell lymphoma” OR “primary cutaneous CD30-positive large T-cell lymphoma” OR “regressing atypical histiocytosis” OR “regressive atypical histiocytosis” OR “primary cutaneous anaplastic large cell lymphoma” OR “lymphogranulomatosis X” OR “angioimmunoblastic T cell lymphoma” OR “cutaneous T-cell lymphoma” OR “cutaneous T-cell non-Hodgkin lymphoma” OR “cutaneous T-cell non-Hodgkin’s lymphoma” OR “cutaneous TCL” OR “primary cutaneous T cell lymphoma” OR “T-cell cutaneous lymphoma” OR “cutaneous T cell lymphoma” OR “aggressive cutaneous T-cell lymphoma” OR “aggressive primary cutaneous T-cell lymphoma” OR “aggressive primary cutaneous T cell lymphoma” OR “CGD-TCL” OR “cgdcl” OR “cutaneous g and d T cell lymphoma” OR “cutaneous g/d T cell lymphoma” OR “cutaneous gamma-delta TCL” OR “cutaneous gamma/delta T cell lymphoma” OR “cutaneous gammadelta T cell lymphoma” OR “cutaneous gd T cell lymphoma” OR “cutaneous gd-TCL” OR “cutaneous GDTCL” OR “cutaneous T cell lymphoma gamma delta subtype” OR “gamma/delta T-cell lymphoma of the skin” OR “PCGD-TCL” OR “pcgdtl” OR “primary cutaneous gamma delta T cell lymphoma” OR “primary cutaneous gammadelta T cell lymphoma” OR “cutaneous gamma delta T cell lymphoma” OR “aggressive epidermotropic CD8+ T-cell lymphoma” OR “aggressive epidermotropic CD8-positive T-cell lymphoma” OR “Berti lymphoma” OR “Berti’s lymphoma” OR “pcaectcl” OR “primary cutaneous aggressive epidermotropic CD8 T-cell lymphoma” OR “primary cutaneous aggressive epidermotropic CD8+ T-cell lymphoma” OR “primary cutaneous aggressive epidermotropic CD8+ T cell lymphoma” OR “cutaneous T-Cell lymphoma/Sezary syndrome” OR “Sezary disease” OR “Sezary erythroderma” OR “Sezary lymphoma” OR “Sezary’s disease” OR “Sezary’s reticulosis” OR “Sezary’s syndrome” OR “Sezarys syndrome” OR “syndrome sezary” OR “Sezary syndrome” OR “angiocentric cutaneous T-cell lymphoma of childhood” OR “HV-like lymphoma” OR “hydroa vacciniforme-like lymphoproliferative disease” OR “hydroa vacciniforme-like lymphoproliferative disorder” OR “hydroa-like cutaneous T-cell lymphoma” OR “hydroa-like lymphoma” OR “hydroa vacciniforme-like lymphoma” OR “indolent cutaneous T-cell lymphoma” OR “indolent primary cutaneous T-cell lymphoma” OR “indolent primary cutaneous T cell lymphoma” OR “cutaneous T-cell lymphoma/mycosis fungoides” OR “granuloma fungoides” OR “mycoses fungoides” OR “mycosis fungoides lymphoma” OR “mycosis fungoides” OR “Alibert Bazin disease” OR “Alibert-Bazin syndrome” OR “Alibert-Bazin type mycosis fungoides” OR “classic MF” OR “classical MF” OR “classical mycosis fungoides” OR “disease, Alibert Bazin” OR “classic mycosis fungoides” OR “folliculotropic MF” OR “mycosis fungoides-associated follicular mucinosis” OR “folliculotropic mycosis fungoides” OR “granulomatous MF” OR “granulomatous mycosis fungoides” OR “granulomatous slack skin” OR “disseminated pagetoid reticulosis” OR “epidermotropic reticulosis” OR “generalized pagetoid reticulosis” OR “Ketron-Goodman” OR “Ketron-Goodman disease” OR “localized pagetoid reticulosis” OR “Woringer-Kolopp disease” OR “pagetoid reticulosis” OR “CD30 LPD” OR “CD30+ LPD” OR “CD30-positive LPD” OR “cutaneous CD30+ T-cell lymphoproliferative disease” OR “cutaneous CD30+ T-cell lymphoproliferative diseases” OR “cutaneous CD30+ T-cell lymphoproliferative disorder” OR “cutaneous CD30+ T-cell lymphoproliferative disorders” OR “primary cutaneous CD30+ T-cell lymphoproliferative disease” OR “primary cutaneous CD30+ T-cell lymphoproliferative diseases” OR “primary cutaneous CD30+ T-cell lymphoproliferative disorder” OR “primary cutaneous CD30+ T-cell lymphoproliferative disorders” OR “primary cutaneous CD30+ T cell lymphoproliferative disorder” OR “LyP” OR “lymphomatoid papulosis” OR “ALCL of the skin” OR “C-ALCL” OR “Ki-1 lymphoma of the skin” OR “primary anaplastic large cell lymphoma of the skin” OR “primary cutaneous anaplastic large T-cell lymphoma” OR “primary cutaneous CD30 positive anaplastic large cell lymphoma” OR “primary cutaneous CD30 positive anaplastic large T-cell lymphoma” OR “primary cutaneous CD30+ ALCL” OR “primary cutaneous CD30+ anaplastic large cell lymphoma” OR “primary cutaneous CD30+ anaplastic large T-cell lymphoma” OR “primary cutaneous CD30+ large T-cell lymphoma” OR “primary cutaneous CD30-positive large T-cell lymphoma” OR “regressing atypical histiocytosis” OR “regressive atypical histiocytosis” OR “primary cutaneous anaplastic large cell lymphoma” OR “SP-TCL” OR “SPTCL” OR “subcutaneous panniculitic T-cell lymphoma” OR “subcutaneous panniculitis like T cell lymphoma” OR “subcutaneous panniculitis T-cell lymphoma” OR “subcutaneous panniculitis-like TCL” OR “subcutaneous T cell lymphoma”

AND

“adverse drug reaction” OR “drug” OR “drug-induced” OR “induced” OR “induction” OR “trigger” OR “triggered” OR “Jak inhibitor” OR “Romidepsin” OR “afatinib synergism” OR “Thiazides” OR “topical calcineurin inhibitors” OR “tacrolimus” OR “pimecrolimus” OR “Methotrexate”

Total results: 20835

Duplicate results: 6804

Remaining results without duplicates: 14031
